# Human risk taking and metabolic state: No credible evidence for desacyl-ghrelin modulation of neural or behavioural effects

**DOI:** 10.1162/IMAG.a.1330

**Published:** 2026-08-06

**Authors:** Steven Geysen, Angela M. Brands, Heidrun Schultz, Julian Koenig, Marc Tittgemeyer, Jan Peters

**Affiliations:** Biological Psychology, Department of Psychology, University of Cologne, Cologne, Germany; Cognitive Neuroscience Lab, Department of Liberal Arts and Sciences, University of Technology Nuremberg, Nuremberg, Germany; Research Group Milestones of Early Cognitive Development, Max Planck Institute for Human Cognitive and Brain Sciences, Leipzig, Germany; Department of Child and Adolescent Psychiatry, Psychosomatics and Psychotherapy, Faculty of Medicine and University Hospital Cologne, University of Cologne, Cologne, Germany; Max Planck Institute for Metabolism Research, Cologne, Germany; Cologne Excellence Cluster on Ageing and Aging Associated Diseases (CECAD), Faculty of Medicine and University Hospital Cologne, University of Cologne, Cologne, Germany; Department of Neurology and Neurosurgery, McConnell Brain Imaging Centre, Montreal Neurological Institute, Modern Diet and Physiology Research Centre, McGill University, Montreal, Canada

**Keywords:** fMRI, hierarchical Bayesian modelling, hunger, probability discounting, risky decision making, sleep deprivation

## Abstract

Risk taking has often been proposed to depend on homeostatic systems. However, empirical evidence remains inconsistent, and the underlying mechanisms remain debated. Changes in risky decision-making, which are associated with homeostatic alterations, may be affected by underlying ghrelin signaling, a peptide hormone that also interacts with dopaminergic functions. The deacylated form of ghrelin has recently received increased attention. Across two studies, we examined whether experimental interventions known to alter desacyl-ghrelin levels influence risky choice in healthy male participants. In study 1, participants underwent a brief fasting period (N =37 ; N =26 for fMRI analyses), whereas in study 2, participants experienced one night of total sleep deprivation (N =40 ; N =36 for fMRI analyses). Risky decision making was assessed using a standard choice paradigm, and behaviour was analysed using computational modelling. Across both studies, we found no credible effects of the experimental manipulations on the proportion of risky choices. Computational modelling indicated that the standard prospect theory (which describes how participants may overestimate small probabilities and underestimate large ones), without accounting for choice repetition, best described the observed behaviour. However, model parameters were not systematically influenced by the experimental manipulations, and changes in desacyl-ghrelin levels were not robustly associated with decision parameters. Functional MRI analyses revealed no effects of state manipulation on neural representations of subjective value or choice in *a priori* defined regions of interest, including the ventromedial prefrontal cortex, ventral striatum, posterior cingulate cortex, anterior cingulate cortex, and anterior insula. Overall, these findings suggest that state-dependent influences on risky decision making, and their modulation by desacyl-ghrelin, may be weaker than previously thought.

## 1 Introduction

Decision making is a fundamental neurocognitive capacity that often depends on processing the uncertainty and risk associated with available choice options (Fox & Poldrack, 2009; Johnson & Busemeyer, 2010; Morelli et al., 2022). To examine how risk is incorporated in the decision-making process, participants are typically asked to choose between options that differ in reward probability and magnitude (Lee et al., 2023; Rachlin et al., 1991). Such risky choice paradigms usually entail an optimal solution. Multiplying the reward magnitude R by the probability P yields an option’s expected value (EV =R×P); the best option has the highest expected value. However, according to *prospect theory* (PT) by Kahneman and Tversky (1979), it is in the apparent simple calculation of expected value that people deviate from optimality. An alternative model that is frequently used to account for suboptimal risky decision making is the *hyperbolic probability discounting model* (HPDM; Green & Myerson, 2004; Mazur, 1986). *Probability discounting* is the reduction of the subjective outcome value as a function of the probability with which it occurs (Kyonka & Schutte, 2018; Rachlin et al., 1991). While HPDM is strong in its simplicity, PT might better account for risk attitudes (Ligneul et al., 2013).

Previous research employing different paradigms to study risky decision making has identified several neural systems involved in risky decision making (Cui et al., 2022; Levy, 2017; Wu et al., 2021). Risk taking is linked to increased activity in regions associated with the neural representation of subjective reward value (Levy, 2017; Panidi et al., 2022, 2024; Seaman et al., 2018; Yao et al., 2023), including the posterior cingulate cortex (PCC), ventromedial prefrontal cortex (vmPFC), dorsolateral prefrontal cortex (dlPFC), and ventral striatum, in particular the nucleus accumbens (NAc; Bartra et al., 2013; Levy et al., 2010; Nachev et al., 2015; Pearson et al., 2011). Moreover, the anterior insula, anterior cingulate cortex (ACC), and dorsomedial prefrontal cortex (dmPFC) are important for conflict monitoring and exhibit activation increases during risk taking (Brown & Braver, 2007; Lv et al., 2021; Von Siebenthal et al., 2020).

Several previous studies linked homeostatic state changes to the extent to which context modulates risk taking. For example, individuals may act more risk averse shortly after having a meal (Symmonds et al., 2010) and make riskier choices when hungry (Levy et al., 2013; Li et al., 2020; Shabat-Simon et al., 2018), perhaps as a survival mechanism to foster exploratory behaviour and prioritise quick food acquisition (Stephens, 1981). Hunger and satiety are the interoceptive reports of energy deficit or surplus, respectively. Rodent models suggest that both are tightly controlled by well-defined neurocircuitry interactions (Beutler et al., 2017; Burnett et al., 2016; Heisler & Lam, 2017; Liu & Kanoski, 2018; Sutton Hickey & Krashes, 2020), including foremost hypothalamic areas (Siemian et al., 2021) and the hippocampus (Davidson et al., 2007; Kanoski & Grill, 2017; Mani et al., 2014), amongst others, which strongly interact with the dopaminergic mesoaccumbens pathway (Cassidy & Tong, 2017; Nieh et al., 2016). Support for these interactions has been observed in human participants as well (Hanßen et al., 2023; Kahn & Shohamy, 2013; Wallner-Liebmann et al., 2010). Hunger is signalled in the hypothalamus (arcuate nucleus and paraventricular hypothalamic nucleus) in rodents (Brüning & Fenselau, 2023; Chen et al., 2015; Heisler & Lam, 2017; Liu et al., 2012). While hunger is operationally difficult to define, physiological proxies linking hunger state to metabolic signals may be relevant in informing state-adaptive processes. To that end we considered ghrelin as a critical peptide which signals the state of hunger, which in turn drives hunger-related behaviour (Anderson et al., 2023; Müller et al., 2015; Zanchi et al., 2017).

The two main circulating forms of ghrelin that hold distinct endogenous physiological roles are acyl-ghrelin and desacyl-ghrelin (Hosoda et al., 2000; Kojima et al., 1999). Both forms of ghrelin generally increase in anticipation of food intake and decrease following consumption, although they may behave differently under specific conditions (Cummings et al., 2001; Delhanty et al., 2012; Deschaine & Leggio, 2022; Foster-Schubert et al., 2008). Ghrelin is mainly produced in the stomach (Kojima et al., 1999; Tschöp et al., 2000) and regulates feeding behaviour, metabolism, and memory. Animal models demonstrated that these signals are sent to the hypothalamus by ghrelin crossing the blood–brain barrier (Banks et al., 2002; Murtuza & Isokawa, 2018; Rhea et al., 2018; Schaeffer et al., 2013) and via the vagus nerve (Date et al., 2006; Wren et al., 2000; Yanagi et al., 2018). In humans, the role of the vagus nerve in ghrelin signalling is less clear (le Roux et al., 2005; Perelló et al., 2022; Yanagi et al., 2018).

Hunger elicits approach and motivation for food-seeking behaviour. Ghrelin is again a key component here since these behaviours are found to be mediated by acyl-ghrelin-induced dopaminergic activity in the VTA and NAc (Abizaid et al., 2006; Al Massadi et al., 2019; Schulz et al., 2023; Stievenard et al., 2017). At the same time, increased dopamine (DA) levels may be linked to increases in risk taking (Rigoli et al., 2016; Rutledge et al., 2015), although the exact nature of this relationship is still unclear (Castrellon et al., 2019). The suspected role of ghrelin in risky choice behaviour is supported by the observation that participants with higher acyl-ghrelin levels scored higher on reward sensitivity and lower on punishment sensitivity (Ralevski et al., 2018), which may be conceptually related to risk taking. In addition, gamblers continue to gamble longer in the face of losses when their acyl-ghrelin levels were higher (Sztainert et al., 2018) and injected acyl-ghrelin reduced sensitivity to losses in women (Pietrzak et al., 2023). While originally thought to be inactive, desacyl-ghrelin is now understood to act independently, often opposing or modulating the metabolic actions of acyl-ghrelin (Delhanty et al., 2012; Deschaine & Leggio, 2022). Most research on risky decision making in humans focused on acyl-ghrelin by demonstrating its role in reward processing that extends beyond food-related rewards (e.g., Pietrzak et al., 2023, 2024). Recent studies suggest that desacyl-ghrelin mechanistically moderates active ghrelin responses, arguably also in the brain (Broglio et al., 2004; Fernandez et al., 2016; Xu et al., 2025). Animal studies found that desacyl-ghrelin interacts with dopaminergic neurocircuitry (Wagner et al., 2017; Witley et al., 2024). In humans, desacyl-ghrelin modulated neural activation in the caudate, an important region for goal-directed behaviour (Kroemer et al., 2013). Goal-directed behaviour needs probabilistic inference and, thus, in this context, we study probability discounting: an effect that so far has been only considered by one other study, which investigated the effect of desacyl-ghrelin in appetite regulation in patients with anorexia nervosa (Bernardoni et al., 2020). Here we assess a more fundamental role by focusing on desacyl-ghrelin and its effects on probability discounting in a healthy control population.

Along similar lines, sleep and circadian rhythms affect cognition (see e.g., Harrison & Horne, 2000; Krause et al., 2017; Lowe et al., 2017). Sleep deprivation shows effects on risky choice behaviour similar to those of hunger, with some studies showing that sleep deprivation is associated with increased risk taking (Brunet et al., 2020; Dickinson et al., 2022; Salfi et al., 2020; Venkatraman et al., 2007; Womack et al., 2013). However, other studies show no effect at all (Mao et al., 2023; Maric et al., 2017; Mullette-Gillman et al., 2015). Sleep deprivation may decrease activity in the thalamus and vmPFC (Menz et al., 2012; Thomas et al., 2000; Venkatraman et al., 2011). Moreover, sleep deprivation may increase general reward processing in the mesolimbic system (which includes the VTA and ventral striatum), decreasing the ability of discriminating between different rewards (Krause et al., 2017).

Similar to the effects of hunger discussed above, effects of sleep deprivation may in part result from changes in the levels of several neuromodulators (Kim et al., 2015). For example, animal models show that adenosine interacts with DA (Basheer et al., 2004; Fuxe et al., 2010), and levels increase following sleep deprivation (Basheer et al., 2004; Elmenhorst et al., 2007; Lazarus et al., 2019). Importantly, sleep deprivation also affects several peptides involved in metabolic functions (Kulkarni et al., 2024; van Cauter et al., 2007). Ghrelin plays a role in the modulation of sleep (Deschaine & Leggio, 2022; Hirotsu et al., 2015), and increased ghrelin levels are linked to the acute effects of sleep deprivation (AlDabal, 2011; Schmid et al., 2008; Taheri et al., 2004), although this association may be weaker than the association between hunger and ghrelin (Soltanieh et al., 2021).

While ghrelin is the main focus of the current study, it is important to note that it is not the only peptide affected by homeostatic state modulations. For example, insulin is influenced by feeding behaviour and can interact with the dopaminergic reward system via the VTA and NAc (Liu & Borgland, 2019; Thanarajah et al., 2019). Insulin levels are found to correlate with better performance under uncertainty (Chang et al., 2016). Another example is leptin, a peptide that is affected by both metabolism (Austin & Marks, 2009; Klok et al., 2007) and restricted sleep duration (Spiegel, Leproult, et al., 2004; Spiegel, Tasali, et al., 2004; van Egmond et al., 2023). Leptin signals energy levels over longer time periods (Ahima & Flier, 2000; Friedman, 2019) and it does not seem to depend significantly on individual meals (Austin & Marks, 2009; Friedman, 2019; Korbonits et al., 1997). Interestingly, leptin does interact with the VTA (Fulton et al., 2006; Geisler & Hayes, 2023; Opland et al., 2010), and higher leptin levels may correlate with more risk-taking behaviour (Chang et al., 2016; Symmonds et al., 2010).

In addition to potential state dependencies, decision making is susceptible to a host of potential biases and choice policies. Perseveration, the tendency to repeat previous actions, is a prominent example. Perseveration is defined as a reward-independent process, which depends only on a participant’s choice history (Bornstein & Banavar, 2023; Miller et al., 2019). This could be based on information from the previous trial only (*first-order perseveration*; FOP) or information spanning a longer period (*higher-order perseveration*; HOP). Several studies have shown that behaviour is better described by models with, rather than without, a perseveration term (Palminteri, 2023; Wiehler et al., 2021). We, therefore, extended the risky choice models with perseveration terms (see Methods) and tested whether such models outperformed their respective traditional versions.

We investigated risky decision making under two state manipulations. Participants performed a risky choice task (Peters & Büchel, 2009) during functional magnetic resonance imaging (fMRI) in two separate within-subjects studies. In study 1, participants performed the task hungry versus sated. In study 2, a different group of participants performed the task once following a single night of total sleep deprivation and once following normal sleep. Behaviour was examined using model-agnostic measures as well as a detailed process-level analysis via hierarchical Bayesian modelling.

Based on previous research (Dickinson et al., 2022; Levy et al., 2013; Pietrzak et al., 2023; Shabat-Simon et al., 2018; Venkatraman et al., 2007, 2011) and considerations outlined above, we predicted participants in both the hungry and sleep-deprived conditions to show more risk-seeking behaviour compared with the sated and normal sleep conditions, respectively. In addition, we investigated whether such potential manipulation effects could be explained by manipulation effects on individual-participant ghrelin levels.

## 2 Methods

We report on two separate but related studies, examining the effect of state modulation on human risk taking. The data collected for study 1 (hunger) have previously not been analysed or published. The data collected for study 2 (sleep deprivation) were part of a previous study for which data from another task had previously been published (Rihm et al., 2019). In the current study, we focused on the probability discounting data from both studies, which have not previously been analysed or published. We preregistered our analyses (https://osf.io/nk7v4) after the data collection.

### 2.1 Participants

In study 1, 37 healthy right-handed men, ranging in age from 19 to 36 years old (M±SD=25.62±3.51), volunteered. The behavioural analysis was done for all 37 participants, the neuroimaging analysis contained the data of 26 participants (M=25.35±2.76 , range  =19−30 ). Seven participants did not have complete fMRI data due to technical issues during scanning. One participant was excluded due to peculiar choice behaviour, making it impossible for the first-level choice model to fit to their imaging data. An additional three participants were excluded due to problems with the analysis of the blood samples.

In study 2, 40 healthy right-handed men volunteered. Their age ranged between 19 and 33 years old (M=25.68±3.66). The behavioural analysis was done for all 40 participants, the neuroimaging analysis contained the data of 34 participants (M=26.12±3.59 , range =19−33 ). Two participants did not have complete fMRI data due to scanner problems. Three participants were excluded due to missing endocrine data. One additional participant was excluded due to a BMI value >25 kg/m².

In both studies, participants performed a battery of different tasks (including the probability discounting task) in two sessions, once in a hungry (study 1) or sleep-deprived state (study 2) and once in a sated state or after a normal sleep, respectively, as a baseline measure. During both sessions, functional MRI was recorded during task performance. Prior to participation, all participants provided written and informed consent, and all study procedures were approved by the local institutional review board (ethics committee of the Hamburg Board of Physicians, codes PV3697 and PV4377).

No a priori power analysis was conducted when the study was planned. We, therefore, ran a post hoc power analysis to determine whether the study was sufficiently powered to detect previously reported effects of metabolic state on risk taking (Levy et al., 2013; Symmonds et al., 2010). The mean effect size across the two studies was 0.35 . According to the *pwr* library (Champely et al., 2020) in R (version 4.5.0 ; R Core Team, 2025), to achieve a preferred power of 0.8 with a type I error of 0.05 , the required sample size is N=33 . We were, therefore, sufficiently powered to detect this effect.

### 2.2 Experimental design

Both studies consisted of three sessions, each scheduled on a different day: one behavioural pretest and two counterbalanced experimental sessions. During the pretest, which took place 2 to 5 days before the first experimental session, participants completed a risky choice task for the calculation of each participant’s *indifference point*, that is, the amount for which participants were indifferent between the reference option and the risky option. This was done by adjusting the amount of the risky option after two successive choices for the same option (reduced after two successive risky choices, increased after two successive safe choices). Indifference points were calculated for each reward probability and used to compute participant-specific trials for the fMRI sessions (see below).

The experimental sessions were separated by 1 week. During the experimental sessions, right before participants entered the MRI scanner to start the task, blood samples were collected to analyse participants’ ghrelin, leptin, cortisol, insulin, and glucose levels.

#### 2.2.1 Study 1

This study used a within-subjects design, with participants either sated or hungry in the experimental sessions. Conditions were counterbalanced across participants. On the day of testing the hungry condition, participants were instructed to have their usual breakfast before 9:00 a.m., skip lunch, and not consume any snacks or caloric drinks. The average time since their last meal was 8 hours and 27 (±43) minutes (range =7 hours 30 minutes – 11 hours). In the sated condition, participants were instructed not to deviate from their standard eating behaviour. The average time since the last meal in the sated condition was 2 hours and 24 (±54) minutes (range =1 hour 10 minutes – 4 hours 55 minutes). On experimental days, participants arrived at the institute at 4:00 p.m. They rated their hunger level (1= not at all hungry, to 7= very hungry) on a visual analogue scale and received instructions for the risky-choice task.

#### 2.2.2 Study 2

This study used a within-subjects design, with participants being either well rested or sleep deprived during the experimental sessions. Conditions were counterbalanced across participants. Participants were initially told they could be in the same condition twice. This was done to prevent participants from preparing for the upcoming condition by sleeping more during the day. The evening before each experimental session, at 8:00 p.m., participants came to the institute and received a standardised meal. In the sleep deprivation condition, participants stayed at the institute and were kept awake for an entire night. In the normal sleep condition, participants were sent back home with an Actiwatch 2 (Philips Respironics) and the instruction to sleep normally. The average sleep duration was 6 hours and 44 (±56) minutes. In both conditions, participants were asked to not consume any food or caloric drinks after the standardised meal. The data were collected the following morning between 7:30 a.m. and 9:30 a.m. For more details on the experimental design of study 2, see Rihm et al. (2019).

### 2.3 Risky choice task

In both studies, participants performed a variant of the probability discounting task described by Peters and Büchel (2009). On each trial, participants were presented with a hypothetical monetary reward and the probability of receiving it. The reward magnitude (R) was sampled from a Gaussian distribution with the indifference point of the participant as mean and ranged between €20.50 and €80 . The magnitude was presented alongside one of six different reward probabilities (P∈{0.17, 0.28, 0.54, 0.84, 0.96, 0.99}). This risky option was compared with the safe reference option, which was fixed to €20 guaranteed for the duration of the task. The safe option was not presented on the screen. Participants used an MRI-compatible response box to indicate which of the presented options they preferred.

**Fig. 1.**
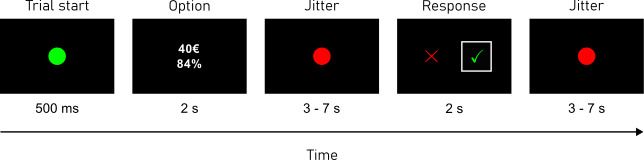
Schematic Illustration of Example Trial. Participants $\left(N_{1}=37,N_{2}=40\right)$ on each trial $\left(T_{1}=96,T_{2}=48\right)$ chose between a safe option (€20 with 100% ) and a risky option presented on the screen. The reward magnitude for the risky option ranged between €20.50 and €80 . Reward probabilities ranged between 17% and 99% (see Methods section). Adapted from Peters and Büchel (2009).

The average duration of each trial was 14 seconds. A new trial was indicated with a green dot, shown for 500 ms . Next, we presented the risky option for 2,500 ms , followed by a red dot (between 3 and 7 seconds, drawn from a uniform distribution). Then, a red cross (safe option) and green checkmark (risky option) appeared on either side of the screen. A box appeared around the selected option for 2 seconds. At the end of each trial, another red dot was presented for 3 to 7 seconds. See Figure 1 for an example trial. Participants were told that a random trial from both experimental sessions would be selected to determine their payout. If they chose the risky option in this trial, the gamble would be played out and participants would receive either the larger reward or nothing. If they chose the safe option, they received €20 .

The two studies differed in the number of trials per condition. In study 1, participants completed 96 trials per condition (total duration ≈22 minutes). In study 2, participants completed 48 trials per condition (total duration ≈11 minutes).

### 2.4 Blood sampling

Blood samples were collected in BD P800 tubes (BD Biosciences, Heidelberg, Germany) filled with a K_2_EDTA anticoagulant for ghrelin and glucose plasma concentration determination and in Sarstedt Serum Gel Monovettes (Sarstedt, Nümbrecht, Germany) for leptin, cortisol, and insulin serum concentration determination. Plasma samples were immediately centrifuged for 10 minutes at 1.2 g at 4°C. The supernatant was immediately pipetted off and stored at −80 °C. Serum samples soaked for at least 45 minutes in the gel solution, and were centrifuged for 10 minutes at 2.0 g at room temperature, and the supernatant was pipetted off and stored at −80 °C. All hormone analyses were conducted by the LADR laboratory in Geesthacht, Germany. Desacyl-ghrelin was analysed with an immunoassay from Bertin Pharma (Paris, France), leptin with a sandwich enzyme-linked immunosorbent assay (ELISA) from DRG (Marburg, Germany), cortisol and insulin with an electro-chemiluminescence immunoassay (ECLIA) method from Roche (Basel, Switzerland), and glucose with a photometric AU 5800 from Beckmann Coulter (Krefeld, Germany). For more details, see Rihm et al. (2019). We used desacyl-ghrelin levels (referred to as ghrelin) for the analyses.

### 2.5 Functional MRI data acquisition

Neuroimaging data were obtained using a Siemens Magnetom Trio 3T whole-body scanner with a 32 -channel head coil. For the functional images, we used single-shot echoplanar imaging and simultaneous multiband acquisitions (slice acceleration factor 2) with TR=2,260 ms, TE=30 ms , number of slices =60 , flip angle =80° , and voxel size =1.5×1.5×1.5 mm .

### 2.6 Behavioural data analysis

We performed exploratory analyses to check whether the experimental manipulations were successful. Similarly, we counted lapses to verify the effectiveness of the sleep deprivation. The proportion of risky choices was used as a model-agnostic index for risk-taking propensity. For each comparison and for each study, we used Bayesian paired samples t-tests to analyse the evidence in favour of an increase in the experimental conditions. This was done in R using the *BayesFactor* library (Morey et al., 2024) with the default priors (Cauchy~(0,0.707). To control for the effect of subjective hunger on the proportion of risky choices, we compared a Bayesian linear model with the hunger rating and condition as predictors of the proportion of risky choices, to a model with only condition as predictor. Both models included a random intercept for the participants. The proportion of risky choices was then further examined depending on the reward probability with a Bayesian ANOVA. The reported Bayes factors (BF± proportional error estimate) were interpreted following the guidelines proposed by Jeffreys (1998) and Lee & Wagenmakers (2013).

### 2.7 Computational modelling

To quantify potential effects on latent subcomponents of risk taking, we set up and compared several cognitive computational models. These are described in the following section and differed in terms of the valuation component (e.g., prospect theory versus hyperbolic probability discounting) and the choice component describing how these subjective values guide participants’ decisions (e.g., models with first-order-perseveration (FOP) versus higher-order perseveration (HOP) versus no perseveration). See Table 1 for an overview of the model space.

**Table 1.** Overview model space.

Model	Free parameters	Equation
Baseline model		
First-order perseveration (FOP)	β	Equation 6
Higher-order perseveration (HOP)	α,β	Equation 6
Prospect theory model	δ,γ,β	Equations 2, 3
Prospect theory with FOP	δ,γ,β	Equations 2, 7
Prospect theory with HOP	δ,γ,α,β	Equations 2, 7
Hyperbolic probability discounting model	k,β	Equations 3, 4
Hyperbolic discounting model with FOP	k,β	Equations 4, 7
Hyperbolic discounting model with HOP	k,α,β	Equations 4, 7

The different models and their free parameters that were compared during the analyses. The underlined model provided the best fit to the behavioural data, based on the WAIC scores (see Supplementary Fig. S7 for the model comparison).

#### 2.7.1 Prospect theory model

The PT model posits that the subjective value $SV_{t}$ of the risky option at trial t is calculated by multiplying the reward magnitude $R_{t}$ with the subjective weight of the reward probability $w\left(P_{t}\right)$:

$$SV_{t}=R_{t}\times w\left(P_{t}\right).$$ (1)

The subjective weight of the reward probability is calculated as

$$w\left(P_{t}\right)=\frac{\delta P_{t}^{\gamma }}{\delta P_{t}^{\gamma }+{\left(1-P_{t}\right)}^{\gamma }}.$$ (2)

Here, the free parameter δ represents the attractiveness of risk and indicates the height of the weighting function. The sensitivity to probabilities γ indicates the degree of curvature of the probability weighting function (Goldstein & Einhorn, 1987; Kahneman & Tversky, 1979; Lattimore et al., 1992; Ligneul et al., 2013; Tymula et al., 2023).

The subjective value and the reward magnitude of the safe option (€20) are then used to estimate the probability of selecting the risky option following the Bernoulli distribution $p_{risky}\;\sim \;Bernoulli\left(\text{logit}\left(\sigma_{t}\right)\right)$ where the standard deviation $\sigma_{t}$ is derived from

$$\text{logit}\left(\sigma_{t}\right)=\beta_{M}\left(SV_{t}-20\right),$$ (3)

with higher $\beta_{M}$ indicating higher stochasticity (i.e., random exploration), and lower values depict value-focused behaviour (i.e., more exploitation).

All models include the Bernoulli distribution to estimate the probability of selecting the risky choice option based on the resulting subjective values.

#### 2.7.2 Hyperbolic probability discounting model

The hyperbolic probability discounting model (Green & Myerson, 2004; Johnson et al., 2020; Kyonka & Schutte, 2018; Mazur, 1986; Rachlin et al., 1991) calculates $SV_{t}$ of $R_{t}$ with

$$SV_{t}=\frac{R_{t}}{1+\exp\left(k\right)\theta_{t}},$$ (4)

where $\theta_{t}$ is the odds against winning the gamble of the risky option and is derived according to

$$\theta_{t}=\frac{1-P_{t}}{P_{t}},$$ (5)

$P_{t}$ is the reward probability. The discount rate k (modelled here in log-space) quantifies how rapidly subjective values change as a function of probability and thus represents the participant’s risk preference. Greater values of k reflect a steeper discounting of value over probability, and, therefore, greater risk aversion.

#### 2.7.3 Prospect theory model with perseveration

Since we expected that accounting for choice repetition could further improve model fit, we added a HOP term, as proposed by Miller et al. (2019), to the PT model (Equations 1 and 2). This HOP term tracks the choice history in a matrix H of size O×T (number of options × number of trials). The matrix was initialised with 0 and is updated at each trial, according to

$$H_{t+1}=H_{t}+\alpha \left(v_{t}-H_{t}\right),$$ (6)

with α as step-size parameter depicting the extent to which previous actions further in the past are updated, and $v_{t}$ is a contrast vector, of which all elements are 0 except for the one corresponding to the selected option at trial t.

To estimate the probability of selecting the risky option, $\sigma_{t}$ of the Bernoulli distribution included the weighted HOP matrix, resulting in

$$\text{logit}\left(\sigma_{t}\right)=\beta_{M}\left(SV_{t}-20\right)+\beta_{H}H_{t},$$ (7)

where scaling parameters $\beta_{M}$ and $\beta_{H}$ control the weight of the model and perseveration term, respectively.

#### 2.7.4 Hyperbolic probability discounting model with perseveration

The same principle is applied to the HPDM. The perseveration term is the same as in Equation 6. The only difference is the calculation of $SV_{t}$ in Equation 7 as it is following the hyperbolic probability discounting model (Equation 4).

For both models with a perseveration term, we had both FOP and HOP versions. The FOP term is a restricted case of HOP, where the step-size parameter α is fixed to 1 instead of being able to vary between 0 and 1. In other words, the FOP term took only the previous trial into account for the calculation of the repetition strength, while the HOP term used all previous trials for the calculations.

#### 2.7.5 Baseline models

The models with a value function were compared with two baseline models, consisting of only a perseveration term. This was either the FOP term or the HOP term.

#### 2.7.6 Model fit and comparison

The different models were fitted using hierarchical Bayesian parameter estimation in RStan (version 2.26.22 ; Stan Development Team, 2023). In a first step, all models were fitted to data from each condition separately, resulting in separate group-level distributions per condition and subject-level parameters. Prior distributions for group-level means were set to be normally distributed (μ=0,σ=5), truncated at 0. To determine the best-fitting model, we compared their widely applicable information scores (WAIC; Watanabe, 2010). Here, lower scores indicate a superior fit. For each condition, in both studies, the PT model without perseveration term had the lowest WAIC score and was, therefore, regarded as best fitting model to the data (see Supplementary Fig. S7).

In a second step, we fitted data from both conditions per study using an adapted version of the PT model (Equation 2), to directly account for condition effects (*shifts*). In order to estimate these effects, we adapted the model so that, for each free parameter, an additional parameter was added that accounts for parameter changes due to the experimental manipulation. In case of the subjective probability weight, this results in

$$w\left(P_{t}\right)=\frac{\left(\delta +c_{t}\times \delta_{s}\right)\times P_{t}^{\gamma +c_{t}\times \gamma_{s}}}{\left(\delta +c_{t}\times \delta_{s}\right)\times P_{t}^{\gamma +c_{t}\times \gamma_{s}}+{\left(1-P_{t}\right)}^{\gamma +c_{t}\times \gamma_{s}}}.$$ (8)

Here, $c_{t}$ depicts a binary indicator which equals 0 for the baseline condition and 1 otherwise. This way, the shift parameters (e.g., $\delta_{s},$ and $\gamma_{s}$) account for the change in parameter estimates due to the condition. For each model, we ran 4 chains with 2,000 samples, half of which were discarded as warmup.

To examine the effects of fasting and sleep deprivation on the different parameters, we focused on the posterior distributions of the group-level parameter derived from the best fitting model. To this end, we provided the 85% and 95% highest density intervals (HDI) and $dBF_{10}$ from Bayesian paired samples t-tests to quantify such potential effects.

### 2.8 Ghrelin analysis

We used Bayesian paired samples t-tests to analyse the evidence in favour of increased ghrelin levels in the experimental conditions (hungry or sleep deprived) compared with the baseline conditions. Similarly, the effect of ghrelin on the proportion of risky choices was analysed by comparing a Bayesian linear model with ghrelin and condition as predictors of the proportion of risky choices, to a model with only condition as predictor. Both models included a random intercept for the participants.

To analyse the effects of ghrelin on the parameters of the PT model, we allowed each model parameter to vary according to the difference in ghrelin levels between experimental conditions. For this we added a ghrelin compound value to each parameter which accounts for the difference in ghrelin levels between the conditions. Using δ as example, its ghrelin compound value $\delta_{g}$ is calculated as

$$\delta_{g}=\Delta_{g}\times \lambda_{\delta },$$ (9)

$\delta_{g}$ contains the difference in ghrelin between conditions $\Delta_{g}$ and the ghrelin coefficient $\lambda_{\delta }$ for the model’s parameter, in this example δ. The same is done for $\gamma_{g}$ and $\beta_{g}$. This resulted in the final PT model where the subjective probability weight (Equation 2) is

$$w\left(P_{t}\right)=\frac{\left(\delta +c_{t}\times \left(\delta_{s}+\delta_{g}\right)\right)\times P_{t}^{\gamma +c_{t}\times \left(\gamma_{s}+\gamma_{g}\right)\;}}{\left(\delta +c_{t}\times \left(\delta_{s}+\delta_{g}\right)\right)\times P_{t}^{\gamma +c_{t}\times \left(\gamma_{s}+\gamma_{g}\right)}+{\left(1-P_{t}\right)}^{\gamma +c_{t}\times \left(\gamma_{s}+\gamma_{g}\right)}}.$$ (10)

### 2.9 Neuroimaging analysis

Preprocessing and analyses of neuroimaging data were done with SPM12 (Penny et al., 2006), following the same procedure as Rihm et al. (2019). The first five scans were removed; the remaining images were realigned to the first scan and unwarped to correct for motion. As Rihm et al. (2019), we too skip slice-timing correction. The anatomical scans were coregistered to the mean EPI image and segmented in grey and white matter, and cerebrospinal fluid (CSF). For each participant, and each condition, we computed noise regressors by applying principal components analysis (PCA) on a mask image of both white matter and CSF.

Imaging analysis focused on two effects. First, we examined neural correlates of subjective value effects followed by an investigation of the effects related to risky versus safe choices.

#### 2.9.1 Subjective value effects

The examination of neural correlates of subjective value effects consisted of replication analyses and analyses of the effect of the state conditions.

For the first-level (within-subject) analyses, we used a different general linear model (GLM) in native space for each participant, for each condition separately. These GLMs included a stick function for the risky option onset, the participant’s z-scored subjective value as computed by the best fitting model as parametric regressor, and the squared z-scored subjective value as a second parametric regressor. The PT parameter values required to calculate the subjective values were estimated by fitting the winning model to the subset of participants used in the fMRI analysis and taking the mean values of each participant’s individual estimated parameter distribution. The GLMs additionally contained a regressor modelling error trials (e.g., trials without responses), as well as noise regressors from the PCA as covariates. All regressors in the model, except for the noise regressors, were convolved with the canonical haemodynamic response function. We applied the default high-pass filter (HPF) cut-off of 128 seconds. Contrast images were created for the linear effects of subjective value. We normalised the segmented anatomical images and the contrast images to standard space coordinates, with a voxel size 1.5×1.5×1.5 mm (IXI549Space of SPM12, similar to Montreal Neurological Institute (MNI) space). The contrast images were smoothed with a 6 mm full-width at half-maximum (FWHM) isotropic Gaussian kernel.

During the second-level analyses, we investigated the main effect of subjective value by comparing the mean BOLD responses of both conditions (experimental and baseline condition) with zero using a t-test. We then tested the effects of state dependencies on the aforementioned value effects by comparing the mean BOLD responses of each experimental condition with the mean BOLD responses in the baseline conditions using a paired t-test. For both tests, we corrected for multiple comparisons by using small-volume corrections (p<.05 , familywise error (FWE) corrected) in regions related to value processing. Therefore, we used the region of interest (ROI) mask provided by the Rangel Neuroeconomics Laboratory (https://www.rnl.caltech.edu/resources/index.html). This mask is based on two meta-analyses (Bartra et al., 2013; Clithero & Rangel, 2014) and defines ROIs which correlated positively with reward value (bilateral vmPFC, ventral striatum, PCC, and ACC).

To investigate the effect of ghrelin in each study, we used again two GLMs, one for each study, to correlate the z-scored difference in ghrelin levels between conditions (baseline versus manipulation) with the parametric modulated brain activation. We used the meta-analysis of Schulz et al. (2023; https://identifiers.org/neurovault.collection:13247 ) as mask for small-volume correction. This mask includes the insula, ventral striatum, and hypothalamus amongst others.

#### 2.9.2 Choice effects

To examine effects related to safe versus risky choices, replication analyses and analyses of the effect of the metabolic state conditions were performed. For the first-level analysis in native space, the GLMs (one for each study) included stick functions for the risky option onsets of safe choices and risky choices, a regressor modelling error trials for risky onsets, and noise regressors from the PCA as covariates. All but the noise regressors in the model were convolved with the canonical haemodynamic response function. We applied the default HPF cut-off of 128 seconds. Contrast images were created for the safe and risky option regressors. We normalised the segmented anatomical images and the contrast images to standard space (IXI549Space) coordinates, with a voxel size 1.5×1.5×1.5 mm . The contrast images were smoothed with a 6 mm FWHM isotropic Gaussian kernel.

During the second-level analyses, we performed a flexible factorial model with choice (safe, risky) and condition (baseline, experimental) as within-subject factors to examine state effects on risk-taking mechanisms. We compared the mean activity of risky choices with the mean activity of safe choices to analyse the main effect of choice. For the main effect of the condition, we compared the activity of both safe and risky choice in the baseline condition with the activity of both safe and risky choice in the experimental condition. Finaly, we analysed the interaction of choice and condition. We compared the neural activity related to the different choices in the sated condition with those in the fasted conditions for study 1. For study 2, we compared the choice-related activity in the well-rested conditions with those in the sleep-deprived condition. We restricted our analyses to regions related to risk taking and used, therefore, the different ROIs involved at the choice option onset from the mask of the meta-analysis of Cui et al. (2022) for small-volume corrections. These ROIs include bilateral ventral striatum, right dmPFC, and left middle occipital gyrus.

Next, we looked at the effect of ghrelin in each study. explained by the difference in ghrelin levels between conditions (baseline versus manipulation) on the interaction between condition and choice. We extracted six different difference images from the first-level GLMs for each study: the differences between conditions for safe choices (baseline minus experimental and experimental minus baseline), the differences between conditions for risky choices, the interactions of choice and condition. These difference images where then smoothed (6 mm FWHM) and correlated with the z-scored difference in ghrelin levels between conditions (baseline versus manipulation). We used the meta-analysis of Schulz et al. (2023; https://identifiers.org/neurovault.collection:13247 ) as mask for small-volume correction.

## 3 Results

### 3.1 Behavioural results

**Fig. 2.**
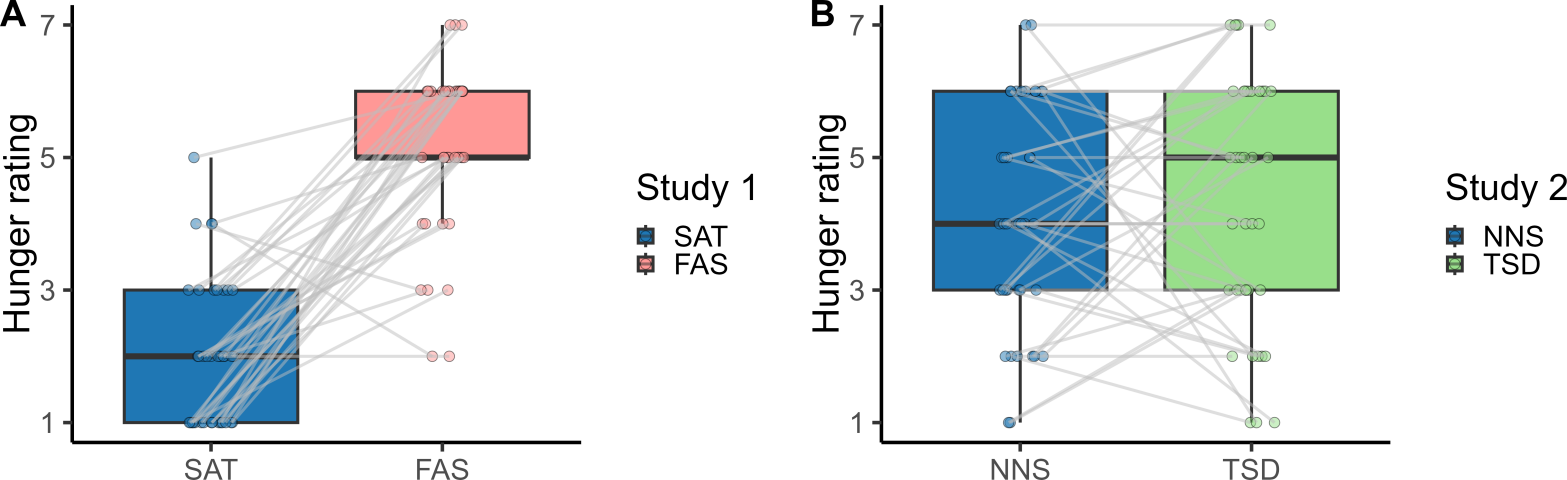
Subjective Hunger Ratings. Participants’ subjective hunger was rated on a 7-point Lickert scale (1= not at all hungry, 7= very hungry). In study 1 (A), the data provided extreme evidence suggesting more subjective hunger in the fasting condition $\left(Mdn_{FAS}=5\right)$ than the sated condition $\left(\text{Md}{\text{n}}_{\text{SAT}}=2,\;\text{BF}>1000\pm 0\%\right)$. In study 2 (B), there was anecdotal evidence against conditional differences in subjective hunger $\left(\text{Md}{\text{n}}_{\text{NNS}}=4,\;\text{Md}{\text{n}}_{\text{TSD}}=5,\text{BF}=0.21\pm 0.05\%\right)$.

Subjective feelings of hunger, rated before scanning, were increased in the fasted condition (FAS; $Mdn_{FAS}=5$ ) compared with the sated condition (SAT; $Mdn_{SAT}=2$ ; Fig. 2A). The data provided extreme evidence in favour of condition differences in subjective hunger ratings in study 1 (BF>1000±0%), suggesting that the hunger manipulation was effective. In study 2, the data provided anecdotal evidence against a difference in subjective hunger ratings between conditions (night of normal sleep, NNS; total sleep deprivation, TSD; $Mdn_{NNS}=4,\;Mdn_{TSD}=5,\;BF=0.21\pm 0.05\%$ ; Fig. 2B). One participant did not rate their subjective hunger; their behavioural and fMRI data were still included in subsequent analyses. Explorative analyses controlling for hunger are presented in the Supplementary Material (Supplementary Figs. S1, S2, S3, and Supplementary Tables S1, S2, S3).

**Fig. 3.**
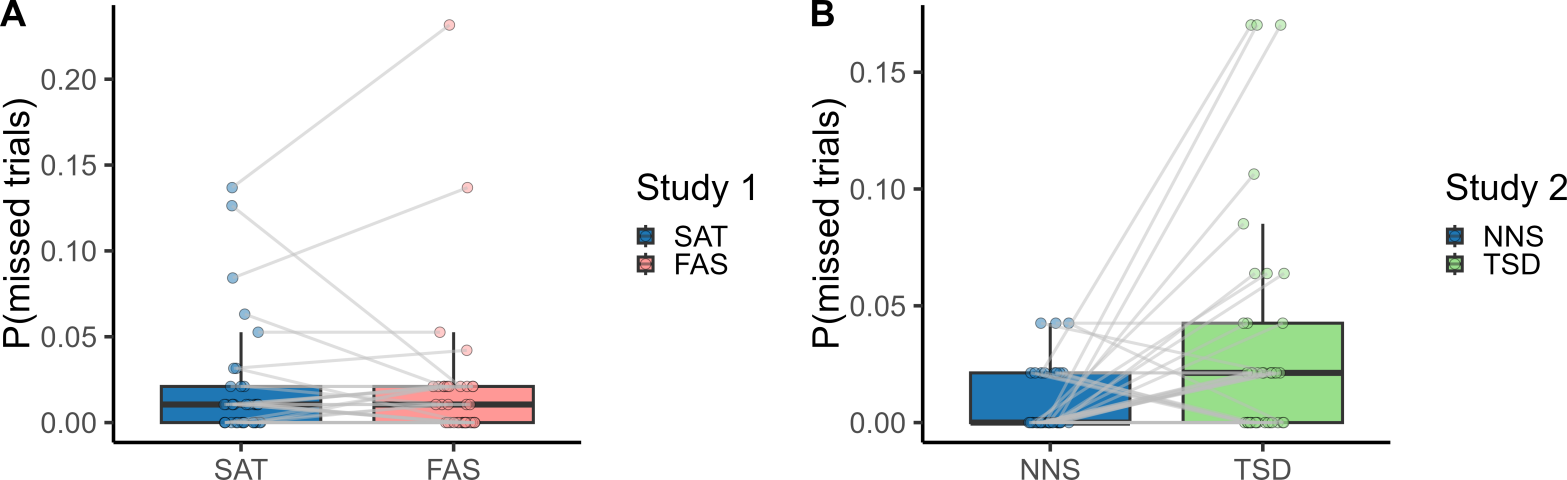
Proportion of Missed Trials. In study 1 (A) there was no difference in missed trials between the sated condition $\left(\text{Md}{\text{n}}_{\text{SAT}}=1.05\%\right)$ and the fasted condition $\left(\text{Md}{\text{n}}_{\text{FAS}}=1.05\%,\text{BF}=0.18\pm 0.05\%\right)$. In study 2 (B), the data provided moderate evidence suggesting participants made fewer lapses after one night of normal sleep $\left(\text{Md}{\text{n}}_{NNS}=0\%\right)$ than one night of total sleep deprivation $\left(\text{Md}{\text{n}}_{\text{TSD}}=2.13\%,\;\text{BF}=9.19\pm 0\%\right)$*.*

To verify the manipulation of sleep deprivation, we compared the number of lapses between conditions. In study 1, the data provided moderate evidence against a difference in missed trials between the sated condition $\left(Mdn_{SAT}=1.05\%\right)$ and the fasted condition. ($Mdn_{FAS}=1.05\%,\;BF=0.18\pm 0.05\%$ ; Fig. 3A). For comparison, in study 2, participants made more lapses after one night of total sleep deprivation $\left(Mdn_{TSD}=2.13\%\right)$ than one night of normal sleep ($Mdn_{NNS}=0\%$ ; Fig. 3B). While the number of lapses after one night of total sleep deprivation is still small, the data provided moderate evidence for an increase in missed trials (BF=9.19±0%), suggesting that the manipulation of sleep deprivation was effective.

**Fig. 4.**
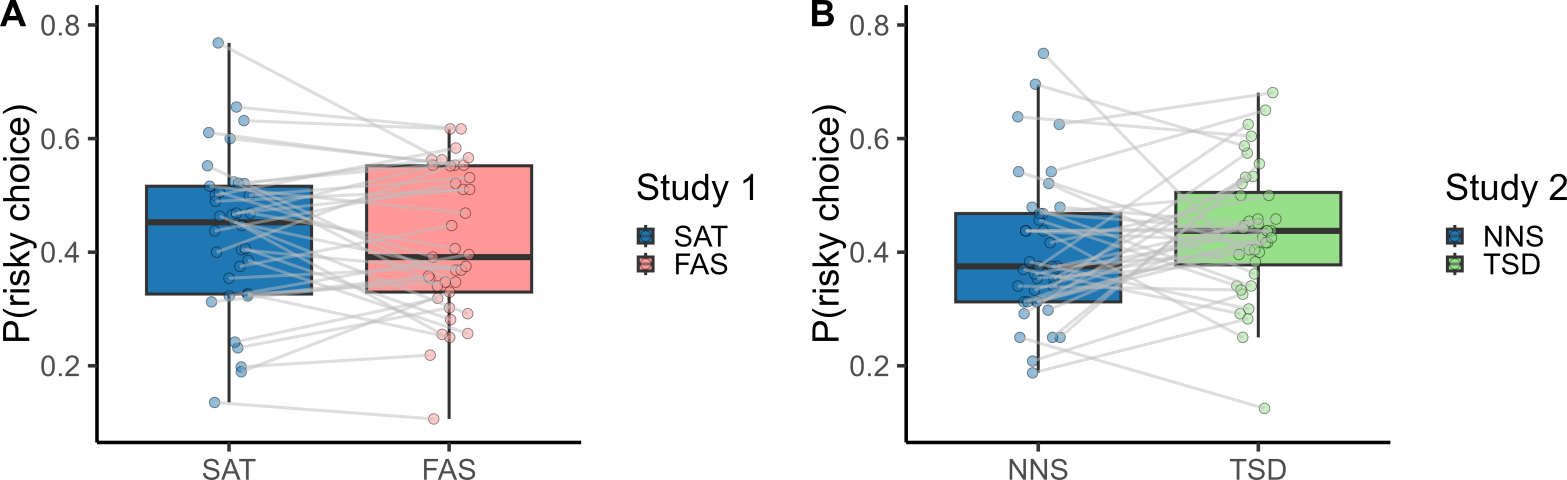
Median Proportion of Risky Choices. In study 1 (A), the median proportion of risky choices was higher in the sated condition ($\text{Md}{\text{n}}_{\text{SAT}}=0.45$ ; blue) than in the fasted condition ($\text{Md}{\text{n}}_{\text{FAS}}=0.39$ ; red). However, the data provided moderate evidence against a significant difference between both conditions (BF=0.31, ±0.04%). In study 2 (B), the median proportion of risky choices was smaller after a night of normal sleep ($\text{Md}{\text{n}}_{\text{NNS}}=0.38$ ; blue) than after one night of total sleep deprivation ($\text{Md}{\text{n}}_{\text{TSD}}=0.44$ ; green). However, the data provided anecdotal evidence against a significant difference between both conditions (BF=0.52±0.03%).

In study 1, the median proportion of risky choices was descriptively higher in the sated condition $\left(Mdn_{SAT}=0.45\right)$ than in the fasted condition ($Mdn_{FAS}=0.39$ ; Fig. 4A). However, the data provided moderate evidence against a significant difference between conditions (BF=0.31±0.04%). In study 2, the data provided anecdotal evidence against condition differences in the median proportion of risky choices ($Mdn_{TSD}=0.44,\;Mdn_{NNS}=0.38,\;BF=0.52\pm 0.03\%$ ; Fig. 4B). While reaction times were not the focus of our analysis, the data provided moderate (study 1; BF=0.28±0.04% ) to strong (study 2; BF=0.18±0.05% ) evidence against a significant difference between the baseline and the experimental conditions. Further analysis of reaction times is given in the Supplementary Material (Supplementary Fig. S4).

Zooming in on the different reward probabilities, Bayesian ANOVA showed for study 1 that there was extreme evidence for an effect of reward probabilities on proportion of risky choices (BF>1000±0.01% ; see Supplementary Fig. S5A) and anecdotal evidence against an effect of condition (BF=0.07±0.35%). There was extreme evidence against an interaction effect of reward probability and condition (BF<1/1000±1.72%). In study 2, there was evidence for an effect of reward probabilities on proportion of risky choices (BF>1000±0.01%) and anecdotal evidence against an effect of condition (BF=0.35±0.07%). There was very strong evidence against an interaction effect of reward probability and condition (BF=0.01±2.48% ; see Supplementary Fig. S5B). For the median reaction times, the data provided moderate evidence for an effect of reward probability in study 2 (BF=4.28±0.16) but not in study 1 (BF=0.29±0%). More details are given in the Supplementary Material (Supplementary Fig. S6 and Supplementary Table S4).

### 3.2 Endocrine results

**Fig. 5.**
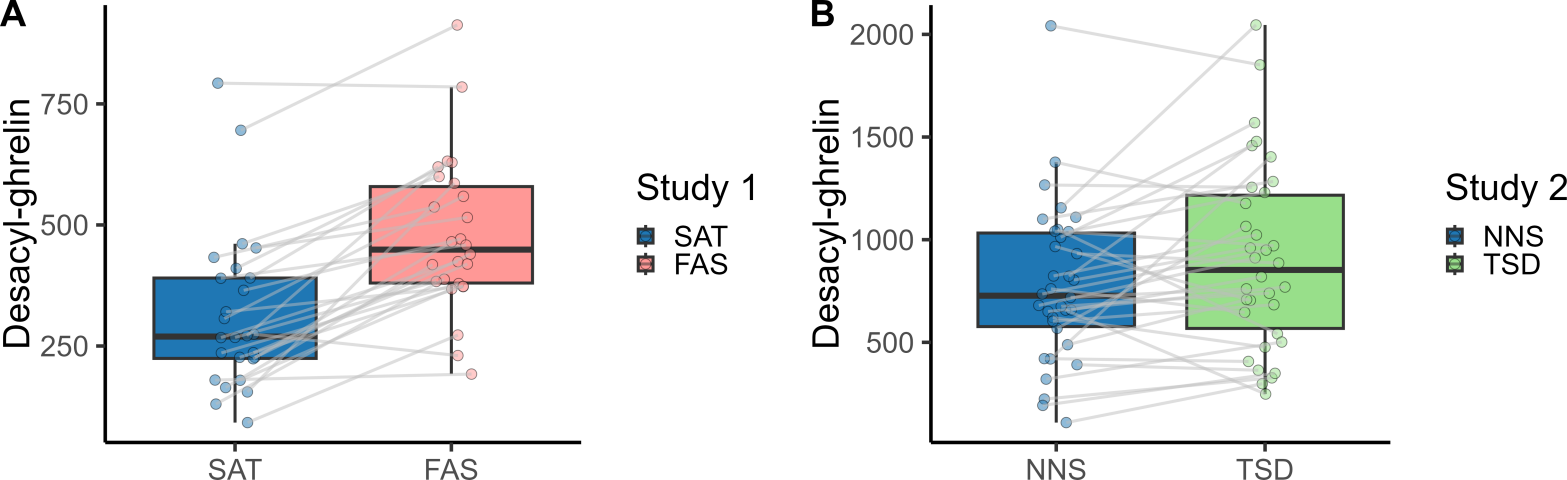
Ghrelin Levels. The data suggested extreme evidence for an increase in ghrelin levels after fasting (A; $\text{Md}{\text{n}}_{\text{SAT}}=269.52,\;\text{Md}{\text{n}}_{\text{FAS}}=449.06,\text{BF}>1000\pm 0\%$ ), while there was inconclusive evidence for a change after a single night of total sleep deprivation (B; $\text{Md}{\text{n}}_{\text{NNS}}=727.14,\;\text{Md}{\text{n}}_{\text{TSD}}=852.68,\;\text{BF}=2.17\pm 0\%$ ). However, note that ghrelin levels after normal sleep were already higher than the sated condition in study 1.

Ghrelin levels per study, subject, and experimental condition are provided in Figure 5. The data suggested extreme evidence for an increase in ghrelin levels after fasting in study 1 $(Mdn_{SAT}=269.52,\;Mdn_{FAS}=449.06,$ BF>1000±0%). In contrast, only anecdotal evidence for an increase in ghrelin levels following sleep deprivation was found in study 2 $(Mdn_{NNS}=727.14,\;Mdn_{TSD}=852.68,$ BF=2.17±0%). Note that for study 2, ghrelin levels in the control condition were substantially greater than control condition ghrelin values in study 1. Rihm et al. (2019) used frequentist analyses, which yields a significant difference in ghrelin levels between normal sleep and a single night of total sleep deprivation. In contrast, Bayesian inference suggested that there is only inconclusive evidence for an increase in ghrelin levels.

**Fig. 6.**
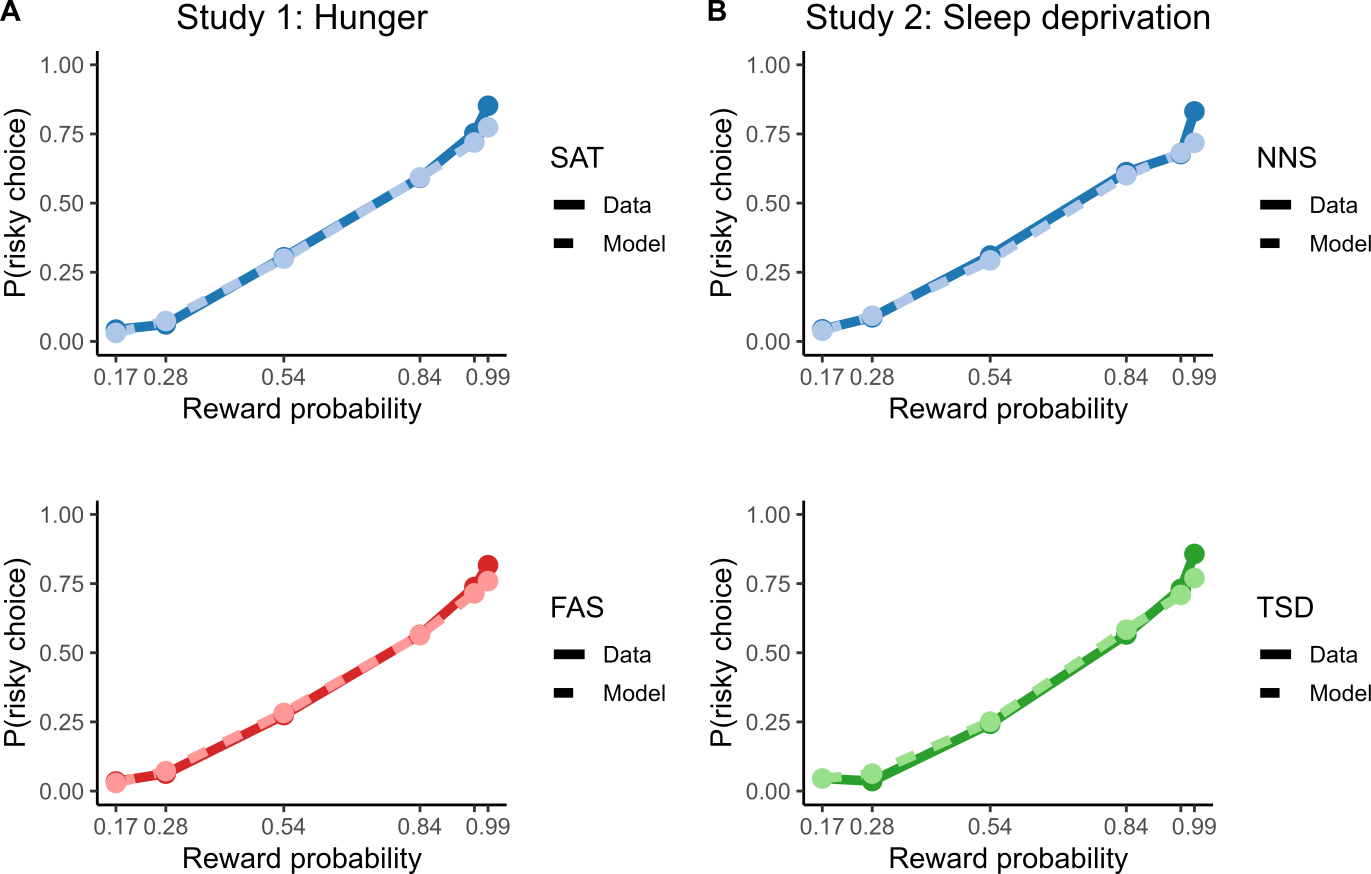
Choice Predictions and Behavioural Data. The probability of selecting the risky option per reward probability (0.17, 0.28, 0.54, 0.84, 0.96, 0.99) for participants (full line) and simulated by the prospect theory model (dashed line) for study 1 (A) and study 2 (B). The model predictions show a large overlap with the behavioural data. It is at the highest reward probability (P=0.99) that the model diverges from the observation.

The group differences of leptin, insulin, cortisol, and glucose are analysed in the Supplementary Material (Supplementary Figs. S12, S16, S17 and S18, respectively).

To analyse the effect of ghrelin on the proportion of risky choices, we compared a Bayesian linear model with ghrelin and condition as predictors of the proportion of risky choices to a model with only condition as predictor. Both models included a random intercept for the participants. The data demonstrated anecdotal evidence against the model with ghrelin as predictor of risky choices (BF=0.44±2.31%) in study 1. Similarly, there was anecdotal evidence against an effect of ghrelin on risky choices (BF=0.6±1.96%) in study 2.

### 3.3 Model-based results

Results from the model comparison via WAIC showed that model variants including a valuation component (either PT or HPDM) were superior to the baseline models (see Supplementary Fig. S7). Model fit was not improved by the inclusion of a perseveration term, neither FOP, nor HOP. Across studies and conditions, the PT model without perseveration provided the best account of the data. To verify that the best-fitting computational model provided a good account of observed behaviour, we performed posterior predictive checks (see Fig. S6). Proportions of risky choices simulated from the PT model showed large overlap with the observed behaviour. This was the case across the full range of reward probabilities, and across studies and experimental conditions.

**Fig. 7.**
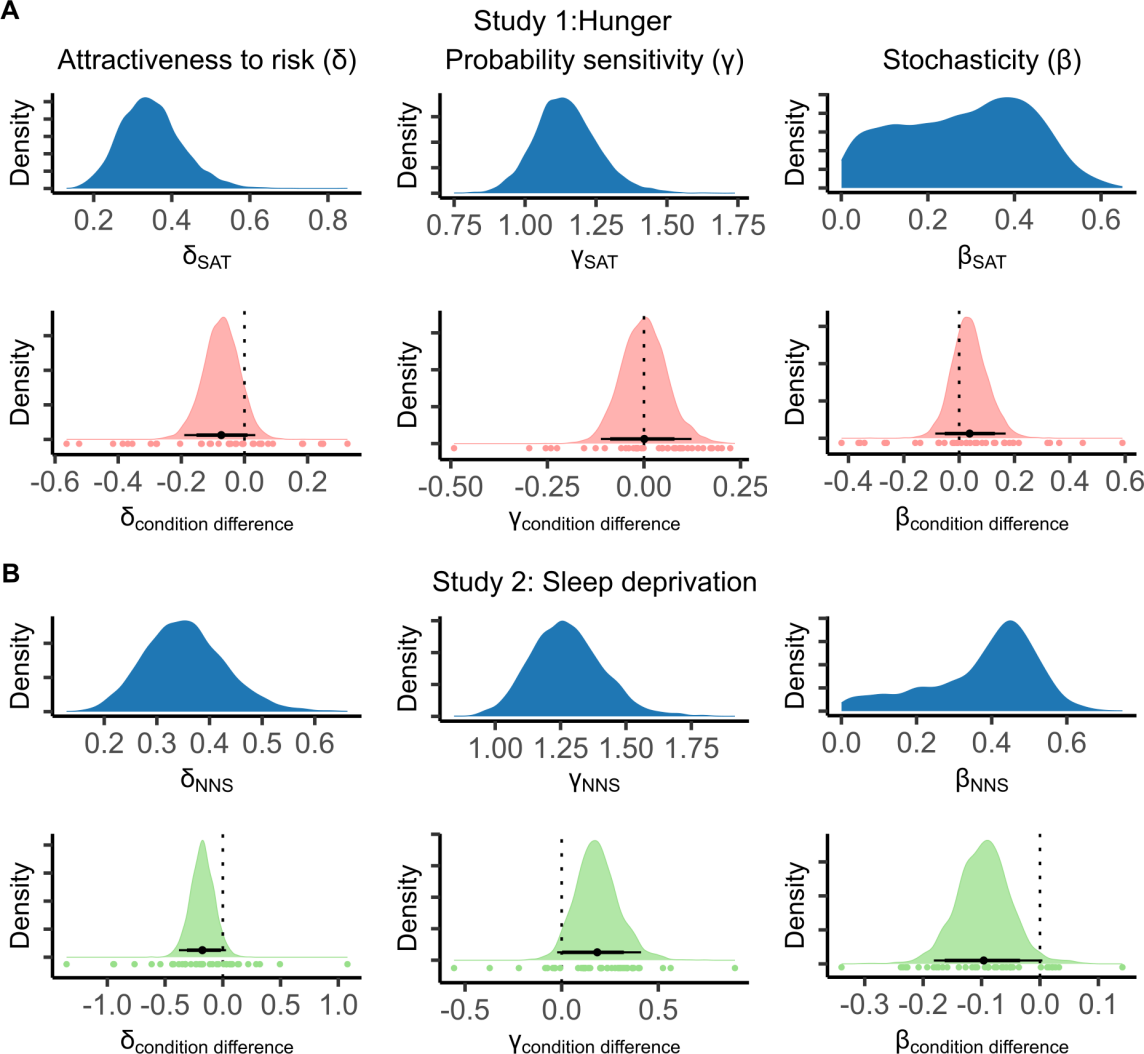
Posterior Distribution of Group-Level Parameters of the Prospect Theory Model. The posterior distributions of the group-level parameters for study 1 (hunger; A) and study 2 (sleep deprivation; B): attractiveness to risk δ, sensitivity to probabilities γ, and policy parameter β. Parameter values of the sated (A) and rested (B) condition are shown in blue, the difference between conditions is shown in red for study 1 and in green for study 2. The horizontal lines represent the highest distribution interval (HDI) of 95% (thin line) and 85% (thick line), with the mean of the distribution as dot. The coloured dots underneath the distribution represent the mean values of individual subjects. See Table 2 for mean and SD values. All parameters, in both studies, did not differ significantly between conditions as shown by the HDI containing zero in each difference distribution.

Next, we examined model parameters, focusing on the best-fitting PT model fitted to the full dataset across experimental conditions (see Equation 8). The posterior distributions of the different parameters and their HDIs are displayed in Figure 7, and numerical values are provided in Tables 2 and 3. In study 1, there were no relevant changes in parameter values between conditions at the group level (see Table 3). The $\overset{↔}{AB}$ HDI contained zero for the difference distributions of each parameter. In study 2, there was a descriptive increase in attractiveness to risk (δ) and the policy parameter (β) after a single night of total sleep deprivation while sensitivity to probabilities (γ) decreased. However, the HDI for the difference distributions of each parameter in study 2 contained zero and we, therefore, conclude that there were no relevant changes in parameter values between conditions at the group level (see Supplementary Tables S5 and S6, and Supplementary Fig. S8 for a replication with the subset of participants used for the fMRI analyses).

**Table 2.** Prospect theory parameter values.

		Study 1	Study 2
δ	Baseline condition	0.35 (±0.08)	0.35 (±0.08)
	Experimental condition	0.31 (±0.08)	0.26 (±0.07)
γ	Baseline condition	1.15 (±0.11)	1.27 (±0.14)
	Experimental condition	1.15 (±0.12)	1.49 (±0.18)
β	Baseline condition	0.28 (±0.15)	0.38 (±0.15)
	Experimental condition	0.32 (±0.16)	0.28 (±0.15)

The mean (±SD) of the blue distributions shown in Figure 7: the estimated parameters for the PT model for the baseline condition (i.e., sated or night of normal sleep) and the experimental condition (i.e., fasted or total sleep deprivation) at the group level. Attractiveness to risk β, sensitivity to probabilities γ, and policy parameter δ.

**Table 3.** Condition differences in prospect theory parameters.

	Mean (±SD)	95% HDI	$dBF_{10}$
Study 1			
Attractiveness to risk (δ)	−0.07 (±0.06)	−0.19, 0.03	0.11
Sensitivity to probabilities (γ)	0 (±0.06)	−0.11, 0.12	1
Policy parameter (β)	0.04 (±0.06)	−0.1, 0.16	2.57
Study 2			
Attractiveness to risk (δ)	−0.18 (±0.1)	−0.38, 0.03	0.05
Sensitivity to probabilities (γ)	0.18 (±0.11)	−0.02, 0.4	23.26
Policy parameter (β)	−0.09 (±0.05)	−0.18, 0	0.02

The difference between the experimental and baseline condition for each study (the coloured distributions in Fig. 7).

**Fig. 8.**
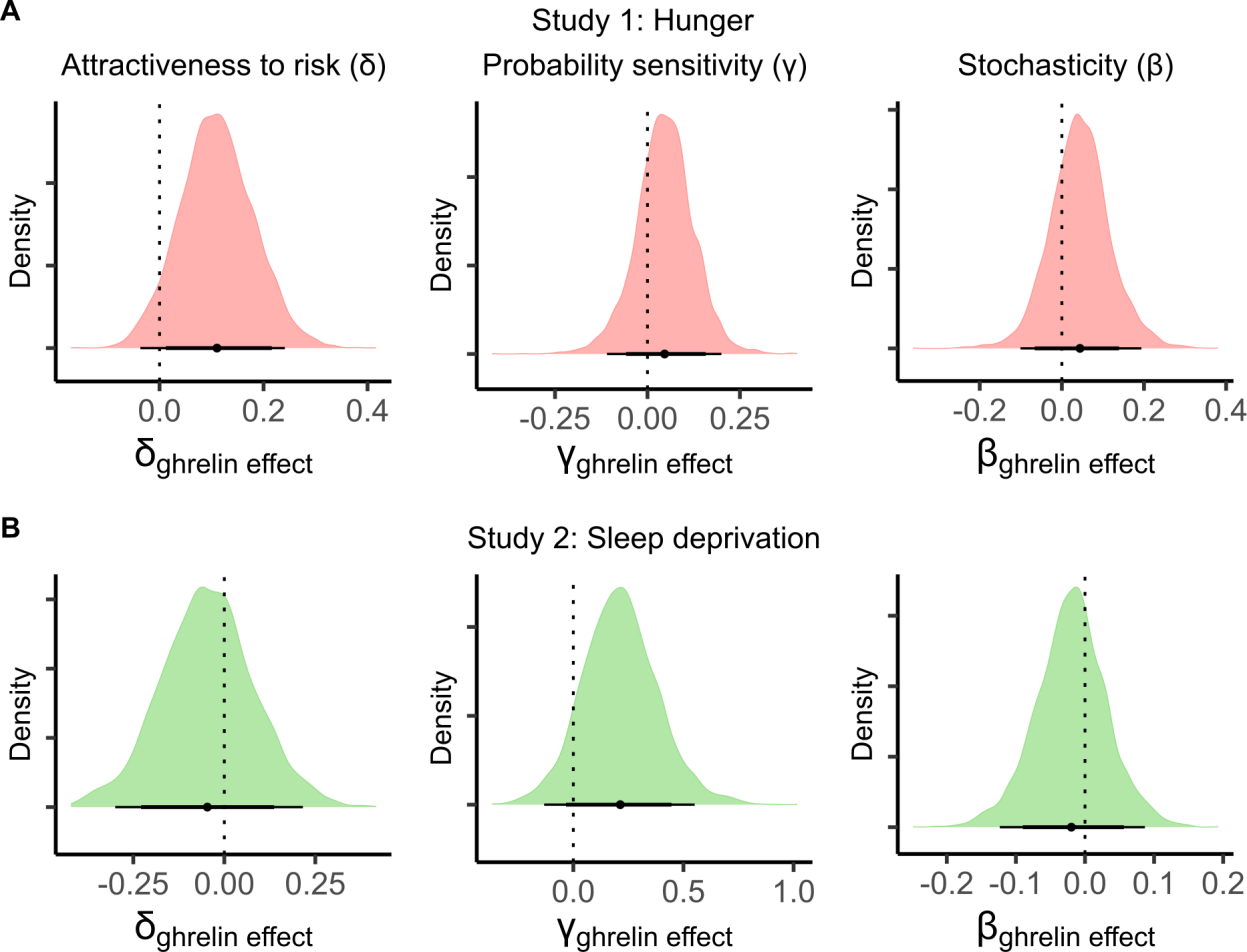
Posterior Distribution of Ghrelin Coefficients for the Parameters of the Prospect Theory Model. The posterior distributions of the ghrelin coefficients for the group-level parameters for study 1 (hunger; A) and study 2 (sleep deprivation; B): attractiveness to risk δ, sensitivity to probabilities γ, and policy parameter β. Zero falls within the highest density intervals (HDI; thick bars: 0.85 , thin bars: 0.95 ) for all parameter in both studies.

To investigate the effect of ghrelin on the parameters, we added ghrelin coefficients to the PT model as described in Equation 10. Note that the sample size is smaller since we only used the data of those participants with endocrine and neuroimaging data (see Supplementary Tables S7 and S8, and Supplementary Fig. S9 for the results of the models’ main parameters). The densities of the different ghrelin coefficients and their HDIs are displayed in Figure 8, their values in Table 4. In both studies 1 and 2, there was inconclusive evidence for effects of ghrelin on any of the estimated parameters of the PT model. Similarly, we performed exploratory analyses to investigate the additional effect of insulin. There was inconclusive evidence for an effect of insulin in both studies (see Supplementary Fig. S13 for the model predictions, the posterior distributions are presented in Supplementary Figs. S14 and S15 and Supplementary Tables S12, S13, S14, and S15).

**Table 4.** Ghrelin coefficients of the prospect theory parameters.

	Mean (±SD)	95% HDI	$dBF_{10}$
Study 1			
Attractiveness to risk (δ)	0.11 (±0.07)	−0.04, 0.24	15.61
Sensitivity to probabilities (γ)	0.05 (±0.08)	−0.11, 0.2	2.86
Policy parameter (β)	0.04 (±0.07)	−0.1, 0.19	2.79
Study 2			
Attractiveness to risk (δ)	−0.05 (±0.13)	−0.3, 0.22	0.55
Sensitivity to probabilities (γ)	0.21 (±0.17)	−0.13, 0.55	8.94
Policy parameter (β)	−0.02 (±0.05)	−0.12, 0.09	0.52

The effect of ghrelin on the estimated parameter values of the PT model presented in Figure 8.

### 3.4 Neuroimaging results

#### 3.4.1 Subjective value effects

**Fig. 9.**
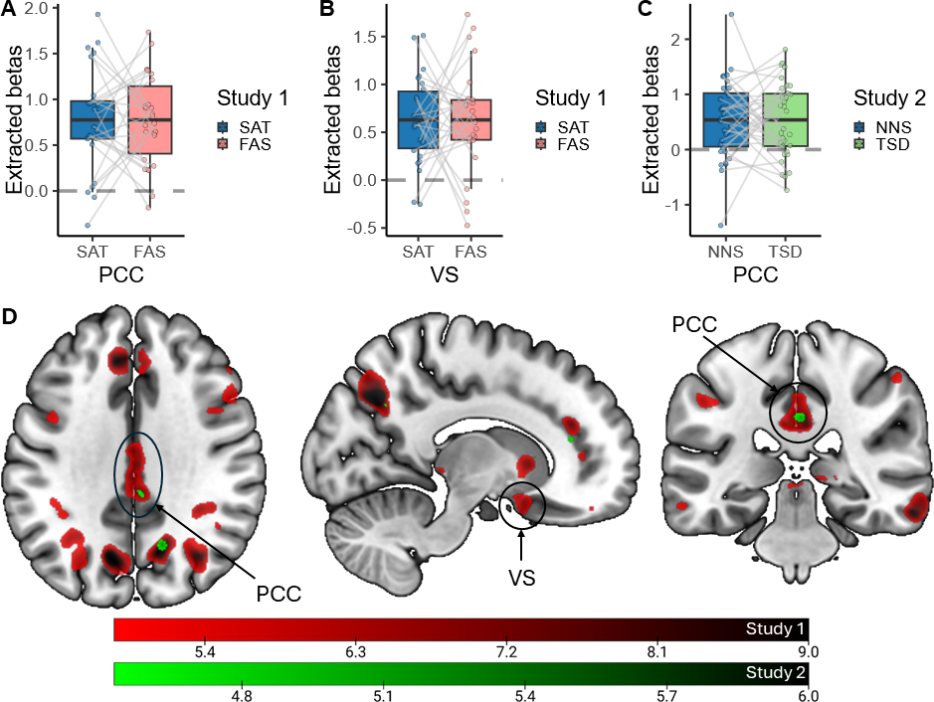
Neural Activity Related to Subjective Value. (A–C) The extracted β-coefficients of subjective value in the right PCC (A; X=2,Y=−32,Z=38 ) and VS (B; 12, 11, −14 ) for study 1, and the right PCC (C; 2, −32, 34 ) for study 2. The β-coefficients support the finding of a main effect of subjective value but no effect of condition. (D) Clusters with significant activity for subjective value for study 1 (red) and study 2 (green). Slices, made on X=13,Y=−32, Z=34 , support the overlap between studies. However, the activity in study 2 is smaller than in study 1. Display threshold: p<.001 uncorrected.

The subjective value was calculated with the mean value of the estimated parameter distributions of each participant. To analyse the activity related to subjective value, we used the ROIs from the mask of the Rangel laboratory, FWE-SVC (see Methods section). Activity of subjective value is in line with previous studies (Fig. 9, see Supplementary Table S9 for an overview of both studies and the MNI coordinates of the regions with statistically significant activity). Study 1 showed a main effect of subjective value in the right PCC, right ventral striatum (NAc), bilateral ACC, and left nucleus caudatus. In study 2, a main effect of subjective value was found in the left nucleus caudatus and ACC, and right PCC. Across both studies, none of the BOLD differences between conditions survived the stricter threshold of FWE-based small volume correction. Additionally, we did not find any region where conditional differences of ghrelin correlated significantly with the neural signatures of subjective value. This, again, was true across both studies. Further support for the lack of effect of ghrelin is presented in the Supplementary Material (Supplementary Table S11 and Fig. S11).

#### 3.4.2 Choice effects

We used the ROIs from the mask of the meta-analysis of Cui et al. (2022), FWE-SVC, for the analysis of neural activity-related choices (see Methods section). Study 1 showed increased activity for risky choices compared with safe choices in the right dlPFC, right inferior parietal gyrus, right inferior temporal gyrus, left anterior insula, and bilateral PCC. Study 2 showed increased activity in the left nucleus caudatus and right dlPFC for risky choices (full results are reported in Supplementary Table S10 and Supplementary Fig. S10). There was no increased activity for safe choices that survived the threshold in either study.

No region showed significant activity related to the interaction of choice (risky versus safe) and condition in our a prior defined ROIs, neither in study 1 nor in study 2. Additionally, we did not find any region where conditional differences of ghrelin correlated significantly with the neural activity of choice or the activity of the interaction of choice and condition. This, again, was true across both studies. Further support for the lack of affect of ghrelin is presented in the Supplementary Material (Supplementary Table S11 and Fig. S11).

## 4 Discussion

In two separate studies, we manipulated desacyl-ghrelin levels via short-term fasting (study 1) or a single night of total sleep deprivation (study 2), to test the prediction that increases in desacyl-ghrelin levels would be linked to increased risk taking (Dickinson et al., 2022; Levy et al., 2013; Pietrzak et al., 2023; Shabat-Simon et al., 2018; Venkatraman et al., 2007, 2011). The effectiveness of the experimental manipulations was confirmed by the increase in subjective hunger ratings in study 1 in the fasting condition compared with the sated condition. In addition, we observed an increase in missed trials after one night of total sleep deprivation compared with one night of normal sleep, a frequent observation in sleep deprivation research (Renn & Cote, 2013; Williams et al., 1959). Moreover, Bayesian analyses revealed that the fasting manipulation successfully elevated desacyl-ghrelin levels, whereas the evidence for an effect of sleep deprivation on desacyl-ghrelin levels was inconclusive (but, see Rihm et al., 2019 for a significant effect using frequentist analysis on the same data).

In contrast with some previous studies that reported an increase in risk taking after fasting (Levy et al., 2013; Shabat-Simon et al., 2018; Symmonds et al., 2010) or sleep deprivation (Brunet et al., 2020; Dickinson et al., 2022; Mckenna et al., 2007; Salfi et al., 2020; Womack et al., 2013), and in line with previously reported null effects (Mao et al., 2023; van Swieten et al., 2023), neither manipulation affected model-agnostic or computational modelling-based measures of risky decision making via changes in desacyl-ghrelin. While we found an effect of subjective hunger ratings on attractiveness to risk and RTs in study 1, there was inconclusive evidence for an effect of desacyl-ghrelin levels on risky choice behaviour or model parameters.

Fasting and sleep deprivation are distinct processes that may affect decision making and risk taking via different routes (Harrison & Horne, 2000; Heisler & Lam, 2017; Krause et al., 2017; Liu & Kanoski, 2018). Importantly, however, both fasting and sleep deprivation elicit effects within the dopaminergic reward network. One possible element that may explain some of the overlapping effects is ghrelin (Kulkarni et al., 2024; Müller et al., 2015; Schulz et al., 2023; Stievenard et al., 2017). VTA and NAc, core regions of the mesolimbic DA neurocircuitry, are modulated by ghrelin (Abizaid et al., 2006; Al Massadi et al., 2019; Schulz et al., 2023; Stievenard et al., 2017). Since ghrelin increases both following fasting and sleep deprivation (AlDabal, 2011; Cummings et al., 2001; Müller et al., 2015; Schmid et al., 2008), it has been hypothesised as a potential mechanism underlying effects on risky decision making (Müller et al., 2015; Schulz et al., 2023; Sztainert et al., 2018). However, we found inconclusive evidence for associations between ghrelin levels and risk taking.

Two main circulating forms of ghrelin are present in the body: acyl- and desacyl-ghrelin (Hosoda et al., 2000; Kojima et al., 1999). It is thought that both types act in an antagonistic manner in rodents (Asakawa et al., 2005; Fernandez et al., 2016) and in humans (Broglio et al., 2004; Delhanty et al., 2012). Both acyl- and desacyl-ghrelin can interact with the dopaminergic reward system, likely via distinct pathways (Broglio et al., 2004; Fernandez et al., 2016; Wagner et al., 2017; Witley et al., 2024; Xu et al., 2025). This interaction suggests a possible effect of desacyl-ghrelin on risky decision making. However, the absence of an effect in our studies is in line with the few others that investigated the role of desacyl-ghrelin on decision making (Bernardoni et al., 2020; Rihm et al., 2019).

To investigate the potential effects of the state-dependent manipulations on the underlying processes, we followed a computational modelling approach. Our model comparison was consistent across studies and experimental conditions and revealed that the PT model without perseveration provided the best account of the data. WAIC scores (Watanabe, 2010) penalise models with more parameters, thus decreasing the likelihood of overfitting (Vandekerckhove et al., 2015). Model comparison was also supported by posterior predictive checks, which confirmed that the best-fitting model accurately reproduced the observed data across reward probabilities and experimental conditions.

In contrast to previous work (Palminteri, 2023; Wiehler et al., 2021), accounting for perseveration did not further improve model fit. One likely explanation is the absence of exploration and exploitation in the current paradigm. Choice repetition may play a greater role in situations where participants need to find an optimal balance between exploration and exploitation (Abir et al., 2024; Lerner, 2020; Miller et al., 2019), for example, during reinforcement learning in volatile environments, perseveration may help to stabilise choice patterns (Ferguson et al., 2023; Wiehler et al., 2021). However, in the present risky choice paradigm, exploration is not required, as all choice-relevant information is provided.

Examination of parameter estimates derived from the best-fitting model allowed us to analyse latent processes involved in risky decision making. Although there was no difference in risky choice behaviour between the baseline and experimental condition, it is possible that similar behavioural patterns can result from different combinations of parameter values. However, in line with the model-agnostic analyses, these analyses revealed little credible evidence for state-dependent effects. These null results were relatively robust, and observed when analysing all participants, only the subset of participants completing the fMRI sessions, or when incorporating ghrelin as a covariate directly in the model. There was also inconclusive evidence for an effect of condition differences in ghrelin levels on model parameters in either study. This adds to the ongoing discussion on metabolic influences on risk taking, by showing that changes in ghrelin levels are unlikely to drive changes in risk taking following two different metabolic manipulations.

The only credible condition effect reflected an increase in choice stochasticity (reduction in the policy parameter β) following one night of total sleep deprivation. This is in line with previous work (Glass et al., 2011; Menz et al., 2012), and confirms that sleep deprivation can lead to increases in choice stochasticity. The small effect of one night of total sleep deprivation on choice stochasticity, but not on risky decision making, could potentially link to the overall decreased activity in the thalamus and PFC after sleep deprivation (Thomas et al., 2000, 2003; Womack et al., 2013), regions related to risky decision making (Donoso et al., 2014; Panidi et al., 2022; Preuschoff et al., 2006). Sleep deprivation is strongly associated with stress and metabolism (AlDabal, 2011; Hirotsu et al., 2015; Kim et al., 2015; Kulkarni et al., 2024), and alters reward processing (Mullin et al., 2013; Rihm et al., 2019; Womack et al., 2013). Such effects might underlie the observed effects on choice stochasticity. Additionally, animal models suggest that adenosine levels increase in the basal forebrain during sleep deprivation (Basheer et al., 2004, 2007; Blanco-Centurion et al., 2006). The basal forebrain is involved in the control of attention (Maness et al., 2022) and increased levels of adenosine lower performance on attention tasks (Christie et al., 2008; Urry & Landolt, 2014). Decreased attention might provide an alternative explanation for the increased choice stochasticity (e.g., Hunt et al., 2018) after one night of total sleep deprivation. Note that, according to the Bayesian analysis, the evidence for increased ghrelin levels between the baseline and experimental conditions was inconclusive in study 2. This would be in line with the idea that other sleep deprivation-related effects (and not ghrelin levels per se) may underlie the observed increases in choice stochasticity.

The present study replicated several core neuroimaging effects. First, across both studies, using an a priori defined ROI approach, we replicated subjective value effects in the ACC, PCC, caudate, and ventral striatum (Bartra et al., 2013; Levy et al., 2010; Nachev et al., 2015; Pearson et al., 2011). Second, using a meta-analysis-based mask (Cui et al., 2022), we replicated risky versus safe option choice effects in the anterior insula in study 1 and the caudate in study 2, in addition to the dlPFC across both studies. However, in line with the behavioural and modelling null results, there were no effects of fasting or sleep deprivation on the neural encoding of subjective value or risky versus safe choices. Nor was there evidence for a modulatory effect of ghrelin on the neural encoding of subjective value or choices.

While we expected increased risk-taking behaviour in the experimental conditions, we are not the first to report a lack of change in risk taking following fasting (van Swieten et al., 2023) or sleep deprivation (Greer et al., 2016; Mao et al., 2023; Menz et al., 2012). It is possible that results from earlier studies may reflect false-positive effects due to small sample sizes (e.g., Brunet et al., 2020; de Ridder et al., 2014). However, mixed results were found across both smaller (Acheson et al., 2007; Brunet et al., 2020) and larger (Mao et al., 2023; Sztainert et al., 2018) sample sizes. Alternatively, differences in manipulation methods might explain the mixed results. Some authors state that sleep deprivation does not affect more complex cognitive processes (Goel et al., 2009; Harrison & Horne, 2000), and one could argue that risky decision making would be one of them. However, studies with partial sleep deprivation over a longer period did find a positive effect on increased risky decision making (Brunet et al., 2020; Maric et al., 2017; Salfi et al., 2020).

Another possible explanation for the inconclusive results in the literature may be related to the paradigm used. We investigated risky decision making using only one specific paradigm, probability discounting. This impedes the generalisability of our results to risky decision making in general. This specificity may help explain the mixed results in the literature. Rather than suggesting a general effect of ghrelin on risky decision making, our results align with the possibility that ghrelin may affect task-specific components of decision making. A key distinction may lie in the nature of uncertainty. Tasks with transparent probability information (e.g., explicitly presented probabilities, as in our paradigm) have consistently failed to show effects of hunger (van Swieten et al., 2023) or ghrelin (Pietrzak et al., 2023), whereas tasks with ambiguous or unknown probability information (where participants must infer risks from feedback) often report increased risk taking when participants fasted (Sztainert et al., 2018; van Swieten et al., 2023) or ghrelin was administered (Pietrzak et al., 2024). This divergence may reflect differences in cognitive demands, particularly the role of learning. In tasks without transparent probability information, participants must learn reward contingencies over time, a process that may be influenced by ghrelin’s known effects on reinforcement learning (van Swieten et al., 2021). In contrast, transparent tasks eliminate the need for learning, possibly rendering insensitive to ghrelin’s modulatory effects. Thus, our findings suggest that ghrelin’s influence on risk taking may be contingent on task structure and the presence of uncertainty or learning demands. Future research should systematically examine how different task parameters (e.g., transparency of probabilities, feedback mechanisms, and learning requirements) modulate the effects of ghrelin and other neuromodulators on decision making.

Similarly, the subjective feeling of hunger and ghrelin signalling of the homeostatic state of hunger (Anderson et al., 2023; Müller et al., 2015) may in part dissociate. Despite the lack of desacyl-ghrelin effects, exploratory analysis suggested that participants were less attracted to risk (δ) in the fasted condition of study 1 when controlling for subjective hunger. In addition, there was a difference in RTs after controlling for subjective hunger. A recent meta-analysis showed that ghrelin and subjective hunger are only moderately correlated and that this association may be mainly driven by acyl-ghrelin (Anderson et al., 2023). Regardless, the different effects of subjective hunger and ghrelin signalling cannot explain the mixed results in the literature (see, for example, van Swieten et al., 2021) and it does suggest to look further into both effects.

Several limitations of the present approach deserve mention. The samples consisted of only male participants, limiting generalisability. We restricted ourselves to exclude sex-specific effects. Ghrelin levels are higher in women (Akamizu et al., 2005; Makovey et al., 2007), and by testing only male participants, we avoid known interactions between ghrelin and estradiol (Butera, 2010; Smith et al., 2022). That is, estradiol has been shown to modulate ghrelin’ effects, particularly by altering the expression of GHSR in the ARC (Frazao et al., 2014). In addition, the different sexes show differences in risk taking (Byrnes et al., 1999; Cornwall et al., 2018; van den Bos et al., 2013). Furthermore, sex differences have also been observed in the effects of sleep deprivation (Acheson et al., 2007; Ferrara et al., 2015; Lim et al., 2022), and ghrelin effects on risk taking (Pietrzak et al., 2023). Future research is needed to continue the investigation of sex differences in ghrelin effects.

The age range of the samples related to young adults, 19−36 years. Acyl-ghrelin levels may decrease with age (Nass et al., 2014). In addition, older people may be more risk averse than younger people (Best & Charness, 2015; Rutledge et al., 2016), although these age effects may be small (Mata et al., 2011). Even so, it is important to consider the specific age range when interpreting these results. Moreover, sample sizes were relatively small (Grady et al., 2021; Poldrack et al., 2017), and future studies would benefit from larger sample sizes. Nevertheless, we replicated several fMRI effects in pre-registered ROIs, for example, with respect to subjective value (Menz et al., 2012; Peters & Büchel, 2009; Seaman et al., 2018; Wang et al., 2023) and risky choice (Cui et al., 2022; Rao et al., 2008), showing sufficient power for the replications of the main effects. It is possible that condition effects are more subtle, and the study may have been underpowered for fMRI analyses to detect the more subtle effects of the experimental conditions. In addition, although group-level results (e.g., related to subjective value) might be stable, fMRI results at the single-subject level may not be suitable for individual difference analyses (Fisher et al., 2018; Fröhner et al., 2019). Such reliability issues of fMRI contrasts may also contribute to the absence of an effect of ghrelin on subjective value in the neuroimaging analysis.

Modelling conclusions are restricted to the models included in our model space. We focused on the basic version of two models that have been successfully applied in previous studies, and posterior predictive checks confirmed that the models accurately reproduced observed choice patterns. However, it is possible that models may not have accurately captured the processes underlying behaviour in some participants (Levy et al., 2013). Therefore, it is possible that ghrelin did have an effect on latent choice variables, which were not accounted for in the models we used in our analysis. Future research may include, for example, versions of the cumulative prospect theory (Fennema & Wakker, 1997; Harrison & Swarthout, 2023). Additionally, since we did not focus on reaction times, the inclusion of drift diffusion models could provide additional information about the underlying choice processes (Lee et al., 2023; Peters et al., 2020) and lead to potential interesting further insights.

We applied two experimental manipulations known to modulate ghrelin levels (AlDabal, 2011; Cummings et al., 2001; Deschaine & Leggio, 2022). Nevertheless, we did find several noteworthy differences in the results between both studies. There was a significant effect of subjective hunger on attractiveness to risk and RTs only in study 1, and participants showed increased stochasticity only in study 2. Moreover, the increase in ghrelin levels after one night of total sleep deprivation (study 2) was only significant when using frequentist statistics (Rihm et al., 2019), whereas the effect of fasting (study 1) was substantially more pronounced. The two manipulations of course differ along several other dimensions (e.g., ghrelin levels were much higher overall in study 2, sleep deprivation has several additional physiological and subjective effects), which complicates the direct comparison of the manipulations. Future research could attempt such direct comparison by, for example, adding a condition in study 2 where participants were allowed to eat during the night of total sleep deprivation. Additionally, ghrelin is affected by the circadian rhythm (Cummings et al., 2001; Kulkarni et al., 2024). However, in an attempt to minimise these circadian effects, data collection occurred at the same time for the different conditions.

Lastly, we only considered desacyl-ghrelin. This is the most prevalent form of circulating ghrelin in our organism (Delhanty et al., 2015; Liu et al., 2008; Wang et al., 2022). However, the focus on this one form of ghrelin limits the generalisation of the results to ghrelin action overall. In addition, ghrelin is not the only peptide potentially affected by the experimental manipulations applied, and several peptides might interact (Heisler & Lam, 2017; Kim et al., 2015; Kulkarni et al., 2024; Narayanan et al., 2010). Leptin is involved in several state-dependent processes and interacts with the VTA (Fulton et al., 2006; Geisler & Hayes, 2023; Opland et al., 2010). While leptin and ghrelin act in opposite directions with regard to energy intake, they do not appear to interact directly with each other (Klok et al., 2007; Perelló et al., 2012). Glucagon-like peptide-1 (GLP-1; Elliott et al., 1993; Krieger, 2020) and liver-expressed antimicrobial peptide 2 (LEAP2; Ge et al., 2018) are two other peptides that behave in the opposite direction of ghrelin (Decarie-Spain & Kanoski, 2021; Ge et al., 2018; Schulz et al., 2023). Recent works suggested that the decreased effect of rewards in relation to desacyl-ghrelin (Kroemer et al., 2013; Witley et al., 2024) might be mediated by GLP-1 (Xu et al., 2025). Adenosine levels are mediated by sleep deprivation (Basheer et al., 2004; Elmenhorst et al., 2007; Lazarus et al., 2019), and may interact with ghrelin (Tullin et al., 2000; Yang et al., 2011). In general, more research is needed to better understand the role of the different peptides and hormones that are affected by hunger and sleep deprivation, how they interact with each other, and how they influence behaviour.

In summary, we report on two independent fMRI studies which tested the degree to which state-dependent changes (in particular ghrelin levels) impacted risky decision making. Participants performed a probability discounting task, either after a short fasting period or one night of total sleep deprivation. There were no effects of the experimental manipulations or increased ghrelin levels on risky choice behaviour or model-based measures. Finally, there was no neural activity related to the experimental manipulation or the increased ghrelin levels. This suggests that the state-dependent influences, in particular from ghrelin, on risky decision making are weaker than previously thought.

## Supplementary Material

Supplementary Material

## Data Availability

The data and the code for this project are publicly available on OSF (https://osf.io/gcjeq).
