# Supplementary material for: Human risk taking and metabolic state: No credible evidence for desacyl-ghrelin modulation of neural or behavioural effects
Source: Imaging Neurosci (Camb). 2026 Aug 6;4:IMAG.a.1330. doi: 10.1162/IMAG.a.1330 (PMC13449932; doi:10.1162/IMAG.a.1330)
Supplement: Supplementary file 1 [file IMAG.a.1330_supp.pdf]

Human risk-taking and metabolic state: No credible evidence for desacyl-ghrelin modulation of neural or behavioural effects: Supplementary material

Steven Geysen[1], Angela M. Brands[1], Heidrun Schultz[2,3], Julian Koenig[4], Marc Tittgemeyer[5,6,7], and Jan Peters[1]

[1] Biological Psychology, Department of Psychology, University of Cologne, Germany

[2] Cognitive Neuroscience Lab, Department of Liberal Arts and Sciences, University of Technology Nuremberg, Germany

[3] Research Group Milestones of Early Cognitive Development, Max Planck Institute for Human Cognitive and Brain Sciences, Leipzig, Germany

[4] Department of Child and Adolescent Psychiatry, Psychosomatics and Psychotherapy, Faculty of Medicine and University Hospital Cologne, University of Cologne, Germany

[5] Max Planck Institute for Metabolism Research, Germany

[6] Cologne Excellence Cluster on Ageing and Aging Associated Diseases (CECAD), Faculty of Medicine and University Hospital Cologne, University of Cologne, Germany

[7] Department of Neurology and Neurosurgery, McConnell Brain Imaging Centre, Montreal Neurological Institute, Modern Diet and Physiology Research Centre, McGill University, Canada

Supplementary Material

Controlling for Subjective Hunger

The reviewers proposed controlling for subjective hunger levels. While this analysis was not preregistered, the remark is valid and we wanted to address it. To control for subjective hunger on the proportion of risky choices, we applied the same method as for controlling for ghrelin. We compared two Bayesian linear models. The baseline model contained condition as predictor of risky choices, the full model contained the subjective hunger ratings and condition. Both models included a random intercept for the participants. Note that there was no subjective hunger rating for one participant in study 2 $\left(N_{Study\ 1} = 37, N_{Study\ 2} = 39\right)$. When controlling for hunger, Bayesian inference suggested that there was moderate evidence against a difference between conditions in study 1 $(BF = 0.13 \pm 2.24\%)$ and study 2 $(BF = 0.08 \pm 1.86\%)$. Using the same method, we analysed the effect of subjective hunger on RTs. The data demonstrated extreme evidence in favour of the model with subjective hunger and condition as predictors of RT in study 1 $(BF > 1000 \pm 2.41\%)$ and anecdotal evidence in study 2 $(BF = 1.11 \pm 2.37\%)$.

We analysed the effect of subjective hunger on the parameters of the winning model, the prospect theory (PT) model, using the same method as for analysing the effects of ghrelin. We added a compound value to each parameter, which accounts for the difference in subjective hunger ratings between conditions:

$$w(P_t) = \frac{\left(\delta + c_t * (\delta_s + \delta_h)\right) * P_t^{\gamma + c_t * (\gamma_s + \gamma_h)}}{\left(\delta + c_t * (\delta_s + \delta_h)\right) * P_t^{\gamma + c_t * (\gamma_s + \gamma_h)} + (1 - P_t)^{\gamma + c_t * (\gamma_s + \gamma_h)}}$$

Where e.g., δ_h is the hunger compound value containing the difference in subjective hunger ratings between conditions, and the hunger coefficient for δ (see Equations 9 and 10 for more information).

We fitted this model to the behavioural data and performed posterior predictive checks (Supplementary Figure 1). The proportions of risky choices simulated from the PT model showed large overlap with the observed behaviour. This was the case across all reward probabilities, in both studies, and all experimental conditions.

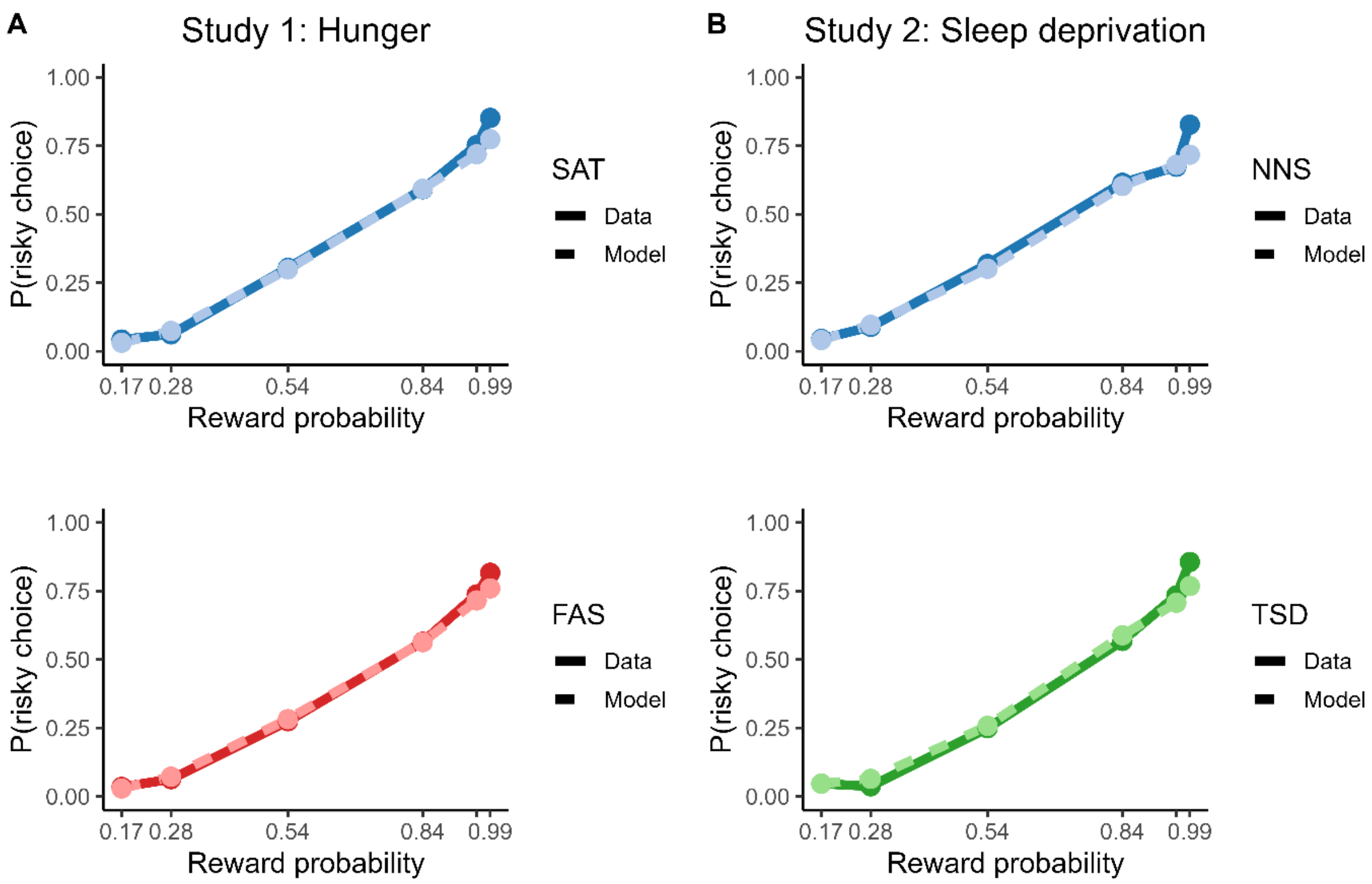

Supplementary Figure 1. Choice Predictions and Behavioural Data. The probability of selecting the risky option per reward probability $(0.17, 0.28, 0.54, 0.84, 0.96, 0.99)$ for participants (full line) and simulated by the prospect theory model (dashed line). The model predictions show a large overlap with the behavioural data.

Next, we investigated the model parameters. The posterior distributions and their HDIs of the different parameters are presented in Supplementary Figure 2, and the numerical values in Supplementary Table 1 andSupplementary Table 2. In study 1, there is very strong evidence for a difference in δ between conditions. The effect of subjective hunger on the model parameters is represented in the posterior distributions of the

coefficients in Supplementary Figure 3 (numerical values in Supplementary Table 3). The posterior distribution of the hunger coefficient of δ is the only one that differed significantly from zero. These results suggests that participants' risk-seeking decreased as their subjective hunger increased.

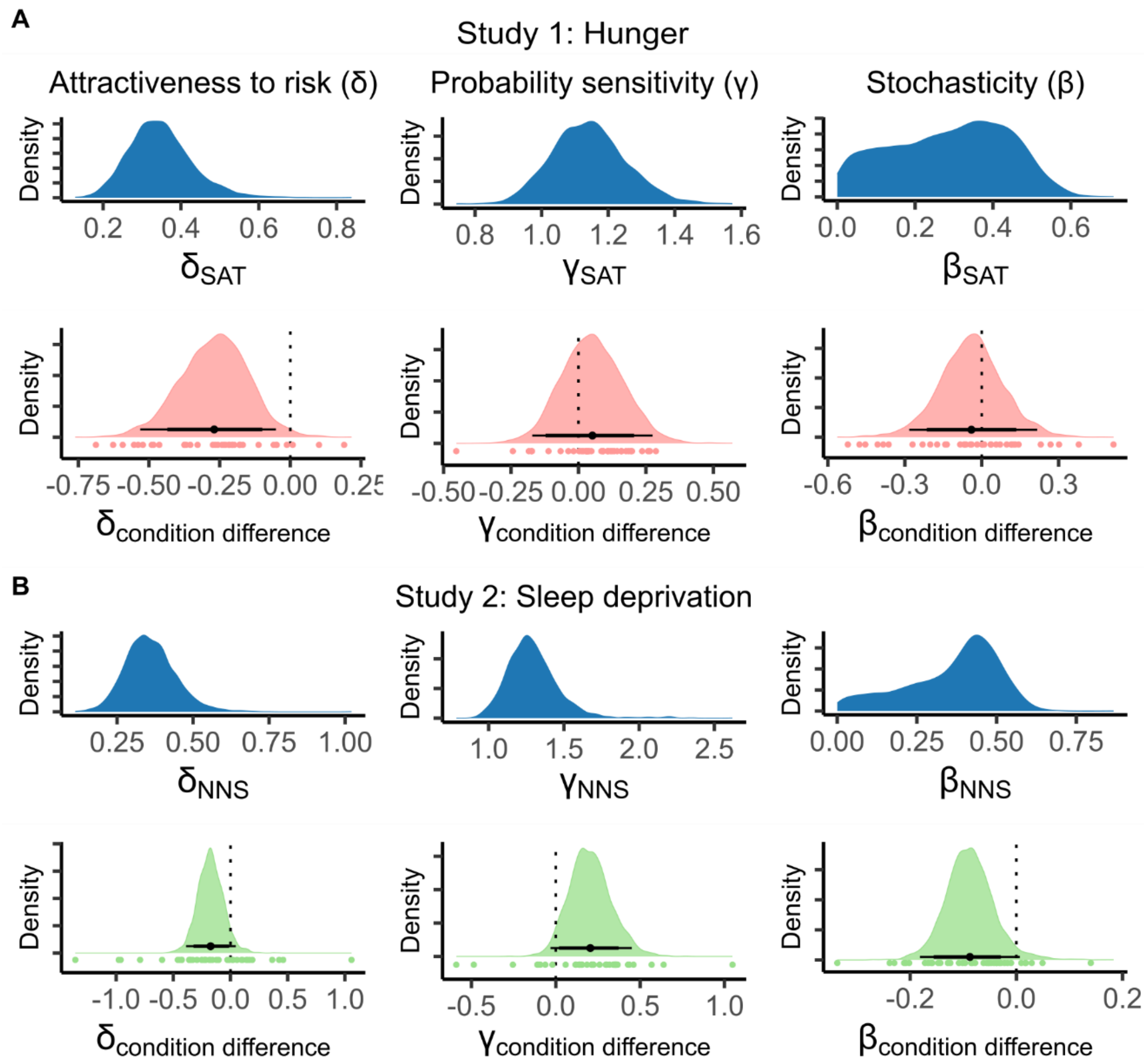

Supplementary Figure 2. Posterior Distributions of Group-Level Parameters of the Prospect Theory Model with Subjective Hunger Coefficients. There was a significant difference between conditions in δ in study 1 as shown by zero falling outside the HDI of the difference distribution. Attractiveness to risk δ, sensitivity to probabilities γ, and policy parameter β.

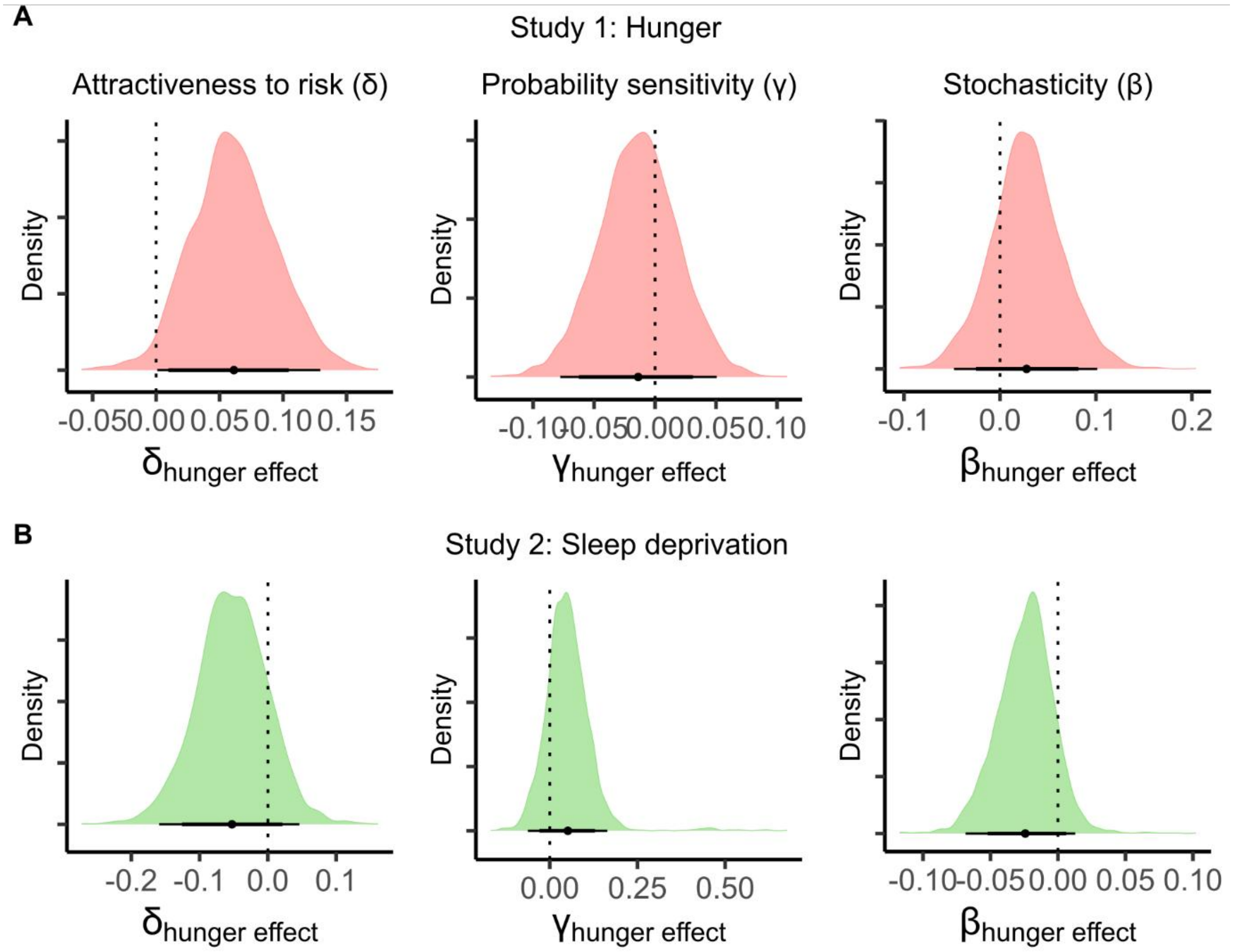

Supplementary Figure 3. Posterior Distributions of Subjective Hunger Coefficients for the Parameters of the Prospect Theory Model. The posterior distributions of the subjective hunger coefficients for the group-level parameters for study 1 (hunger; A) and study 2 (sleep deprivation; B): attractiveness to risk δ, sensitivity to probabilities γ, and policy parameter β. Zero is not captured by the highest density intervals (HDI; thick bars: 0.85, thin bars: 0.95) δ in study 1.

Supplementary Table 1. Prospect Theory Parameter Values with Subjective Hunger Coefficients. Mean ($\pm$SD) of the estimated parameters for the PT model for the baseline condition (i.e., sated or night of normal sleep) and the experimental condition (i.e., fasted or total sleep deprivation) at the group level (blue distributions in Supplementary Figure 2). Attractiveness to risk δ, sensitivity to probabilities γ, and policy parameter β.

		Study 1	Study 2
δ	Baseline condition	$0.35\ (\pm 0.08)$	$0.36\ (\pm 0.08)$
	Experimental condition	$0.22\ (\pm 0.07)$	$0.27\ (\pm 0.09)$
γ	Baseline condition	$1.14\ (\pm 0.11)$	$1.29\ (\pm 0.18)$
	Experimental condition	$1.2\ (\pm 0.17)$	$1.54\ (\pm 0.21)$
β	Baseline condition	$0.29\ (\pm 0.15)$	$0.36\ (\pm 0.15)$
	Experimental condition	$0.25\ (\pm 0.19)$	$0.37\ (\pm 0.15)$

Supplementary Table 2. Conditional Differences of Prospect Theory with Subjective Hunger Coefficients. The difference in posterior distributions between the experimental and baseline condition for each study (the coloured distributions in Supplementary Figure 2).

	Mean (±SD)	95% HDI	dBF_{10}
Study 1			
Attractiveness to risk (δ)	-0.27 (± 0.12)	$-0.53, -0.05$	0.01
Sensitivity to probabilities (γ)	0.05 (± 0.12)	$-0.17, 0.28$	1.98
Policy parameter (β)	-0.04 (± 0.12)	$-0.28, 0.22$	0.57
Study 2			
Attractiveness to risk (δ)	-0.17 (± 0.11)	$-0.39, 0.05$	0.07
Sensitivity to probabilities (γ)	0.2 (± 0.12)	$-0.03, 0.45$	22.96
Policy parameter (β)	-0.09 (± 0.05)	$-0.18, 0.01$	0.04

Supplementary Table 3. Subjective Hunger Coefficients of the Prospect Theory Parameters. The effect of subjective hunger on the estimated parameter values of the PT model presented in Supplementary Figure 3.

	Mean (±SD)	95% HDI	dBF_{10}
Study 1			
Attractiveness to risk (δ)	$0.06\ (\pm 0.03)$	$0, 0.13$	33.94
Sensitivity to probabilities (γ)	$-0.01\ (\pm 0.03)$	$-0.08, 0.05$	0.51
Policy parameter (β)	$0.03\ (\pm 0.04)$	$-0.05, 0.1$	3.54
Study 2			
Attractiveness to risk (δ)	$-0.05\ (\pm 0.05)$	$-0.16, 0.05$	0.19
Sensitivity to probabilities (γ)	$0.05\ (\pm 0.07)$	$-0.06, 0.16$	4.45
Policy parameter (β)	$-0.02\ (\pm 0.02)$	$-0.07, 0.01$	0.12

Reaction Times

The median reaction times (RTs) did not differ between conditions according to a paired Bayesian t-test ($BF = 0.18 \pm 0.05\%$; Supplementary Figure 4). To analyse the effect ghrelin on RTs, we compared a Bayesian linear model with ghrelin and condition as predictors of RTs to a model with only condition as predictor. Both models included a random intercept for the participants. The data demonstrated anecdotal evidence against the model that included ghrelin as predictor of RT ($BF = 0.89 \pm 4.63\%$) in study 1. In study 2, there was strong evidence in favour of an effect of ghrelin on RT ($BF = 32.6 \pm 26.4\%$).

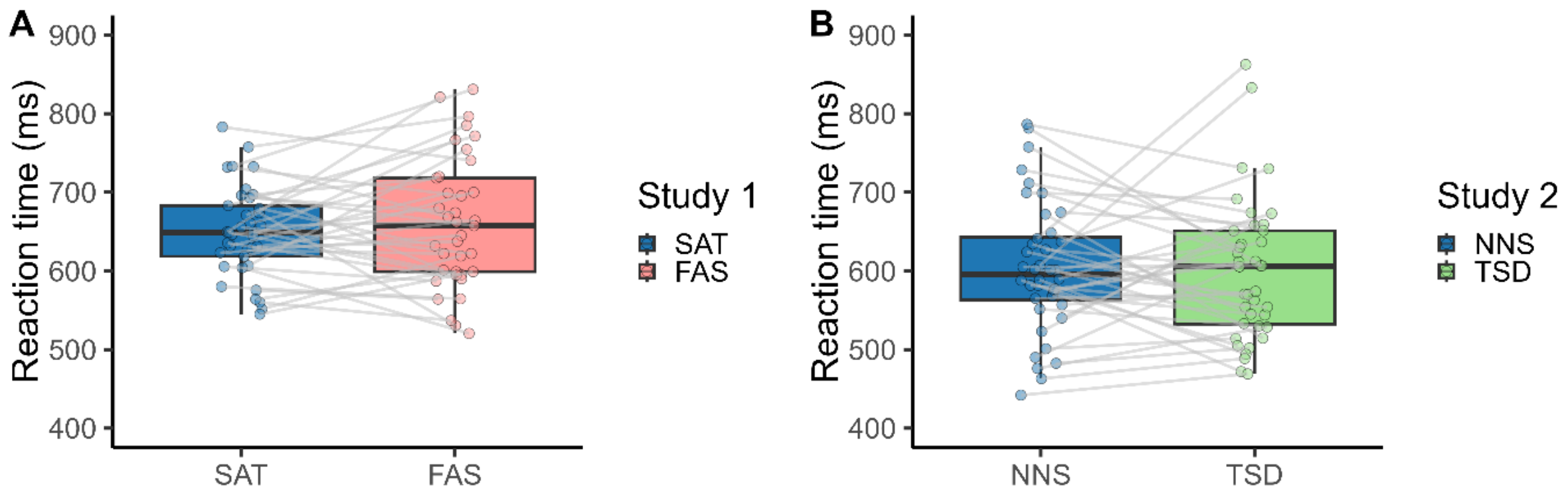

Supplementary Figure 4. Median Reaction Times. Participants in study 1 (A) had faster median reaction times in the sated condition ($Mdn_{SAT} = 649\ ms$; blue), compared to the fasted condition ($Mdn_{FAS} = 657.5\ ms$; red). However, the data provided moderate evidence against a significant difference between both conditions ($BF = 0.18 \pm 0.05\%$). Median reaction times in study 2 (B) were faster after a night of normal sleep ($Mdn_{NNS} = 595.25\ ms$; blue), compared to one night of total sleep deprivation ($Mdn_{TSD} = 605.5\ ms$; green). The data provided strong evidence against a significant difference between both conditions ($BF = 0.18 \pm 0.05\%$).

Effect of Reward Probability

We analysed the proportion of risky choices and median reaction times (RTs) more closely by investigating the effect of reward probability and its interaction with condition. See Supplementary Figure 5 andSupplementary Figure 6 respectively.

We used a Bayesian ANOVA to analyse the effects of reward probability and condition. The results for the proportion of risky choices are presented in the main body of the paper as this was our focus. The results of the median RTs are presented in more detail here. In study 1, there was moderate evidence against an effect of reward probability $(BF = 0.29 \pm 0\%)$ and condition $(BF = 0.14 \pm 0.18\%)$ on median RT. There was extreme evidence against an interaction effect of reward probability and condition $(BF = 6.22 * 10^{-5} \pm 9.23\%)$. In study 2, the data provided moderate evidence for an effect of reward probability on median RT $(BF = 4.28 \pm 0.16\%)$ and anecdotal evidence against an effect of condition on median RT $(BF = 0.08 \pm 0.3\%)$. There was extreme evidence against an interaction effect of reward probability and condition $(BF = 0 \pm 2.38\%)$.

The uncorrected results of Bayesian paired t-tests per reward probability are presented in Supplementary Table 4.

Supplementary Table 4. Bayes Factor per Reward Probability. Results from Bayesian paired t-test ($BF \pm$ proportional error estimate) comparing the difference in the proportion of risky choices from the baseline condition (i.e., sated or night of normal sleep) and the experimental condition (i.e., fasted or total sleep deprivation) to zero, per reward probability. The data did not contain any evidence of such differences. The same is true for reaction times. Uncorrected for multiple comparisons.

Reward probability	0.17	0.28	0.54	0.84	0.96	0.99
Study 1						
Proportion of risky choices						
	$0.08 \pm 0.27\%$	$0.07 \pm 0.31\%$	$0.12 \pm 0.17\%$	$0.08 \pm 0.25\%$	$0.08 \pm 0.27\%$	$0.21 \pm 0.1\%$
Reaction time						
	$0.08 \pm 0.27\%$	$0.07 \pm 0.31\%$	$0.1 \pm 0.2\%$	$0.15 \pm 0.14\%$	$0.24 \pm 0.09\%$	$0.07 \pm 0.31\%$
Study 2						
Proportion of risky choices						
	$0.09 \pm 0.2\%$	$1.01 \pm 0.2\%$	$0.6 \pm 0.03\%$	$0.1 \pm 0.18\%$	$0.21 \pm 0.09\%$	$0.14 \pm 0.13\%$
Reaction time						
	$0.09 \pm 0.2\%$	$0.11 \pm 0.17\%$	$0.31 \pm 0.06\%$	$0.09 \pm 0.2\%$	$0.31 \pm 0.06\%$	$0.1 \pm 0.18\%$

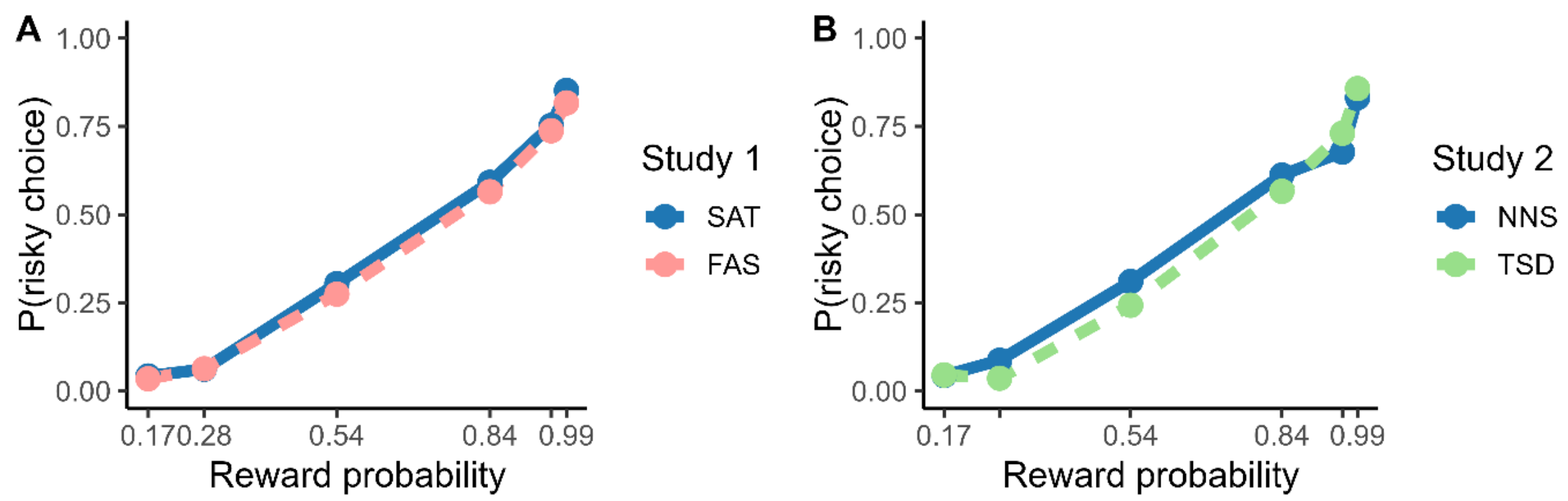

Supplementary Figure 5. Proportion of Risky Choices per Reward Probability. The probability of selecting the risky option per reward probability $(0.17, 0.28, 0.54, 0.84, 0.96, 0.99)$ for each condition.

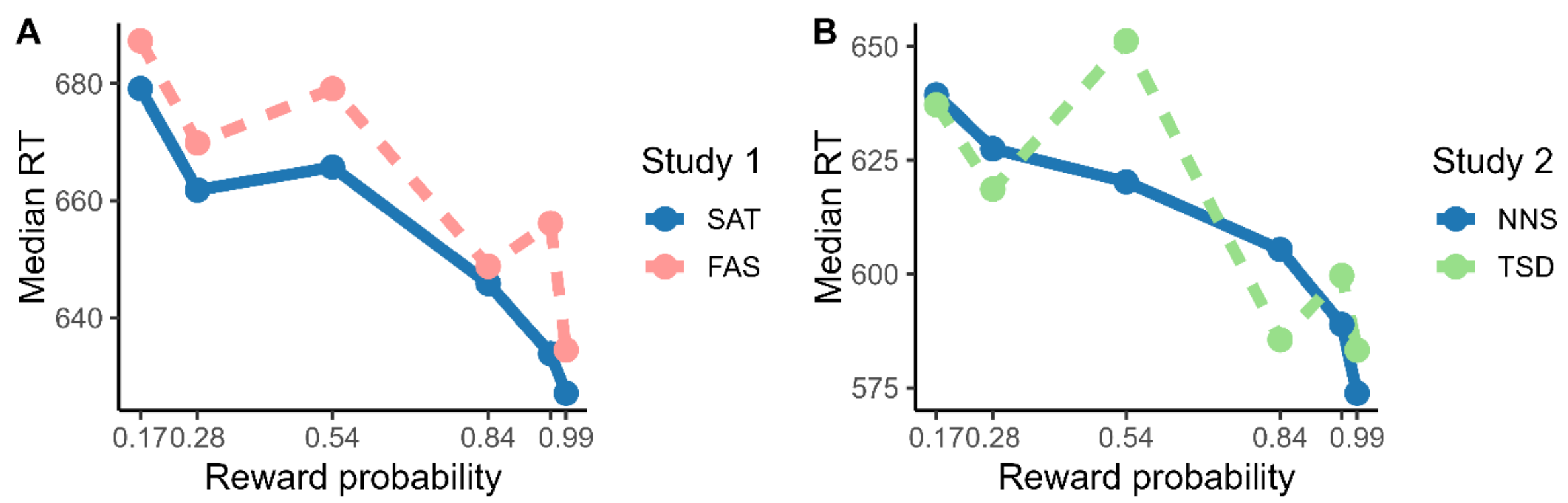

Supplementary Figure 6. Median Reaction Times per Reward Probability. The median reaction times per reward probability $(0.17, 0.28, 0.54, 0.84, 0.96, 0.99)$ for each condition.

Model-Based Results

Model Comparison

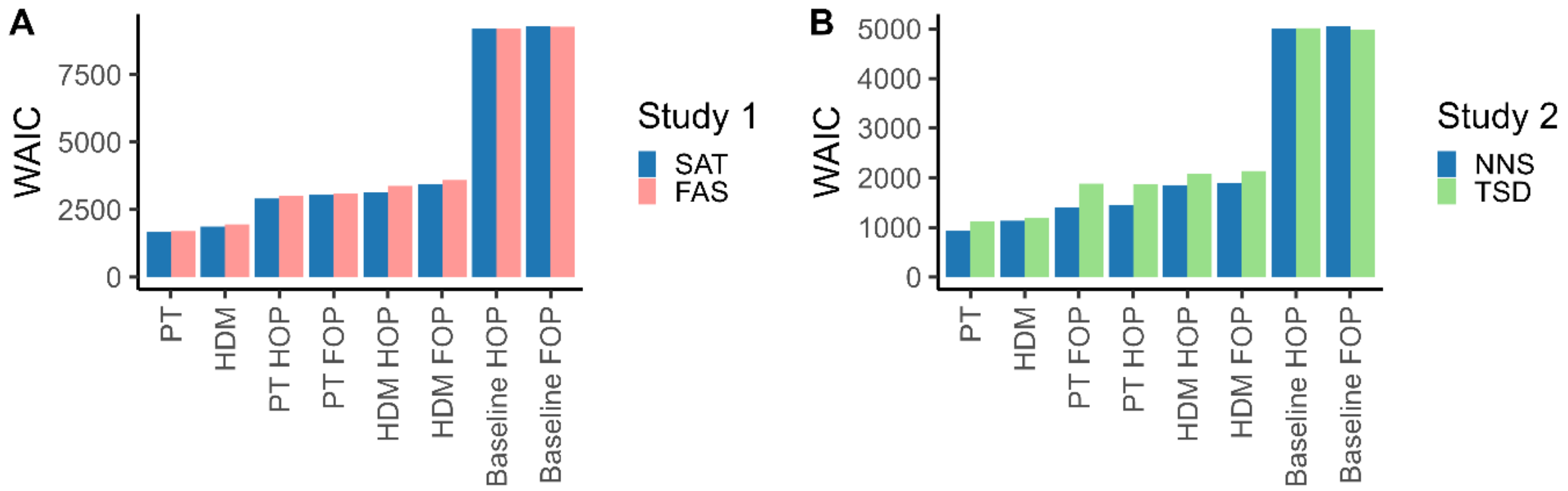

Supplementary Figure 7. WAIC Scores of Model Space. The total WAIC scores for each model per condition. Both the prospect theory (PT) model and hyperbolic probability discounting model (HDM) without perseveration terms performed better than the other models. Since PT had the lowest WAIC score in both conditions, in both studies, we interpreted it as the best fitting model and continued our analyses with this model.

Prospect Theory with fMRI Participants

Here (Supplementary Table 5 andSupplementary Table 6, and Supplementary Figure 8) we report the parameter values of the PT model fitted to the subset of participants of which the neuroimaging data was analysed. The values differ from those in Table 3 and Table 4 due to the different sample sizes. However, the interpretation of the results does not change.

Supplementary Table 5. Prospect Theory Parameter Values with fMRI Participants. Mean ($\pm$SD) of the blue distributions shown in Supplementary Figure 8. Estimated parameters for the PT model for the baseline condition (i.e., sated or night of normal sleep) and the experimental condition (i.e., fasted or total sleep deprivation) at the group level. The model was fitted to the subset of participants of which the neuroimaging data was analysed. Attractiveness to risk δ, sensitivity to probabilities γ, and policy parameter β.

		Study 1	Study 2
δ	Baseline condition	$0.32\ (\pm 0.08)$	$0.34\ (\pm 0.08)$
	Experimental condition	$0.3\ (\pm 0.08)$	$0.24\ (\pm 0.08)$
γ	Baseline condition	$1.2\ (\pm 0.15)$	$1.32\ (\pm 0.17)$
	Experimental condition	$1.25\ (\pm 0.16)$	$1.54\ (\pm 0.22)$
β	Baseline condition	$0.27\ (\pm 0.17)$	$0.35\ (\pm 0.14)$
	Experimental condition	$0.24\ (\pm 0.18)$	$0.24\ (\pm 0.15)$

Supplementary Table 6. Conditional Differences of Prospect Theory with fMRI Participants. The difference between the experimental and baseline condition for each study (the coloured distributions in Supplementary Figure 8).

	Mean ($\pm$SD)	95% HDI	dBF_{10}
Study 1			
Attractiveness to risk (δ)	-0.05 (± 0.06)	$-0.18, 0.08$	0.27
Sensitivity to probabilities (γ)	0.04 (± 0.06)	$-0.08, 0.18$	3.23
Policy parameter (β)	-0.03 (± 0.07)	$-0.17, 0.12$	0.45
Study 2			
Attractiveness to risk (δ)	-0.2 (± 0.12)	$-0.41, 0.05$	0.05
Sensitivity to probabilities (γ)	0.19 (± 0.14)	$-0.08, 0.45$	11.4
Policy parameter (β)	-0.11 (± 0.05)	$-0.2, 0$	0.02

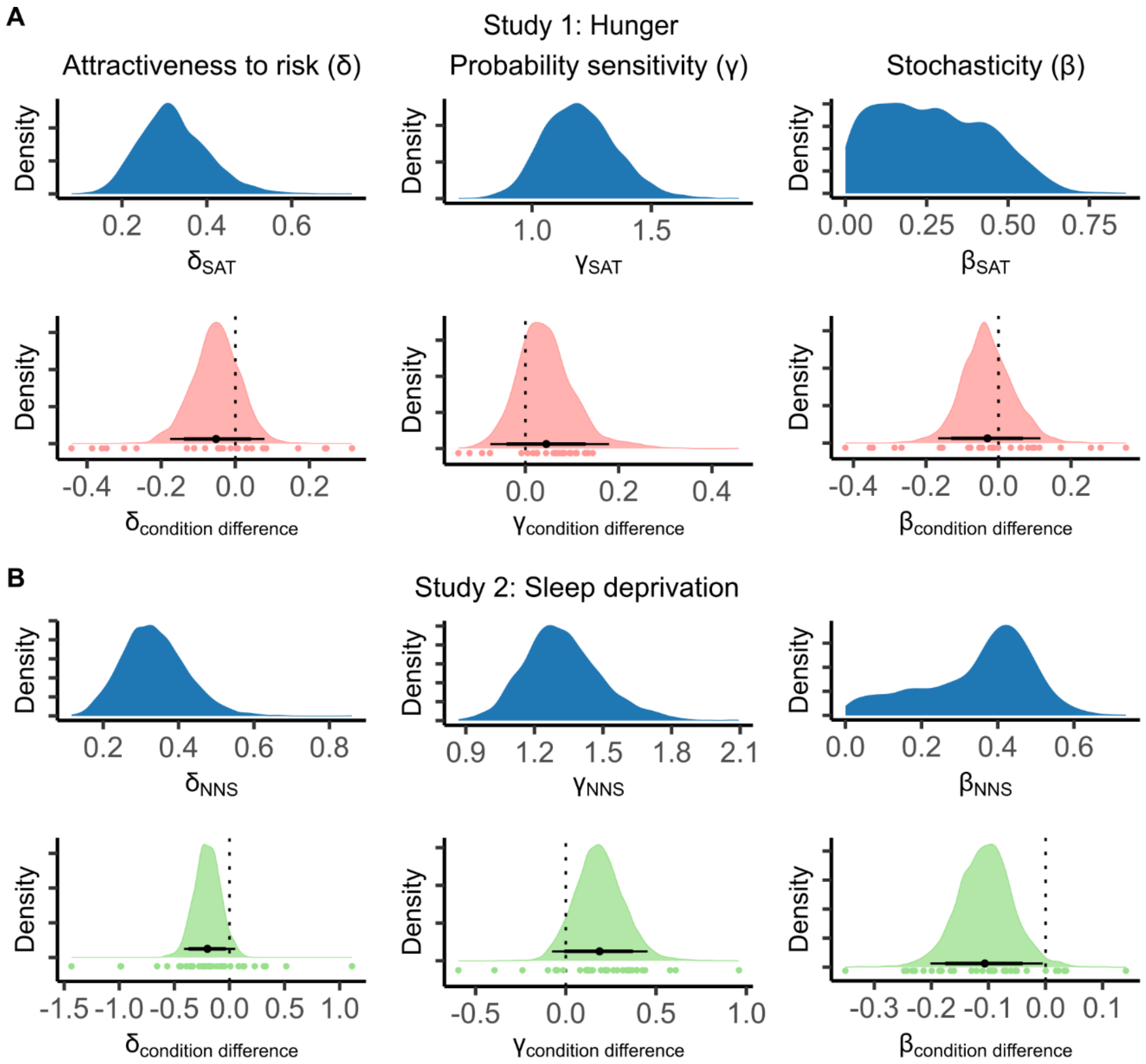

Supplementary Figure 8. Posterior Distributions of Group-Level Parameters of the Prospect Theory Model with fMRI Participants. The posterior distributions of the group-level parameters for study 1 (hunger; A) and study 2 (sleep deprivation; B): attractiveness to risk δ, sensitivity to probabilities γ, and policy parameter β. Parameter values of the sated (A) and rested (B) condition are shown in blue, the difference between conditions is shown in red for study 1 and in green for study 2. The horizontal lines represent the highest distribution interval (HDI) of 95% (thin line) and 85% (thick line), with the mean of the distribution as dot. The coloured dots underneath the distribution represent the mean values of individual subjects. All parameters, in both studies, did not differ significantly between conditions as shown by the HDI containing zero in each difference distribution. Values are reported in Supplementary Table 5 andSupplementary **Table 6**.

Prospect Theory with Ghrelin Coefficients

Here (Supplementary Table 7 and Supplementary Table 8, and Supplementary Figure 9) we report that parameter values of the PT model with ghrelin coefficients fitted to the subset of participants of which the neuroimaging data was analysed. The values differ from those in Table 3 and Table 4 due to the different sample sizes. Additionally, this model contains the ghrelin coefficients for each of the PT parameters. The ghrelin coefficients are reported in the main article (Table 4 and Figure 8).

Supplementary Table 7. Prospect Theory Parameter Values with Ghrelin Coefficients. Mean ($\pm$SD) of the blue distributions shown in Supplementary Figure 9. Estimated parameters for the PT model with ghrelin coefficients for the baseline condition (i.e., sated or night of normal sleep) and the experimental condition (i.e., fasted or total sleep deprivation) at the group level. The model was fitted to the subset of participants of which the neuroimaging data was analysed. Attractiveness to risk δ, sensitivity to probabilities γ, and policy parameter β.

		Study 1	Study 2
δ	Baseline condition	$0.32\ (\pm 0.08)$	$0.34\ (\pm 0.08)$
	Experimental condition	$0.29\ (\pm 0.08)$	$0.23\ (\pm 0.08)$
γ	Baseline condition	$1.21\ (\pm 0.15)$	$1.33\ (\pm 0.17)$
	Experimental condition	$1.27\ (\pm 0.16)$	$1.6\ (\pm 0.22)$
β	Baseline condition	$0.27\ (\pm 0.17)$	$0.35\ (\pm 0.15)$
	Experimental condition	$0.24\ (\pm 0.19)$	$0.24\ (\pm 0.15)$

Supplementary Table 8. Conditional Differences of Prospect Theory with Ghrelin Coefficients. The difference between the baseline and experimental condition for each study (the coloured distributions in Supplementary Figure 9).

	Mean ($\pm$SD)	95% HDI	dBF_{10}
Study 1			
Attractiveness to risk (δ)	-0.05 (±0.06)	$-0.17, 0.08$	0.31
Sensitivity to probabilities (γ)	0.05 (±0.07)	$-0.08, 0.19$	3.49
Policy parameter (β)	-0.03 (±0.08)	$-0.19, 0.12$	0.48
Study 2			
Attractiveness to risk (δ)	-0.22 (±0.12)	$-0.47, 0.02$	0.04
Sensitivity to probabilities (γ)	0.24 (±0.14)	$-0.04, 0.52$	24.55
Policy parameter (β)	-0.11 (±0.05)	$-0.21, -0.01$	0.02

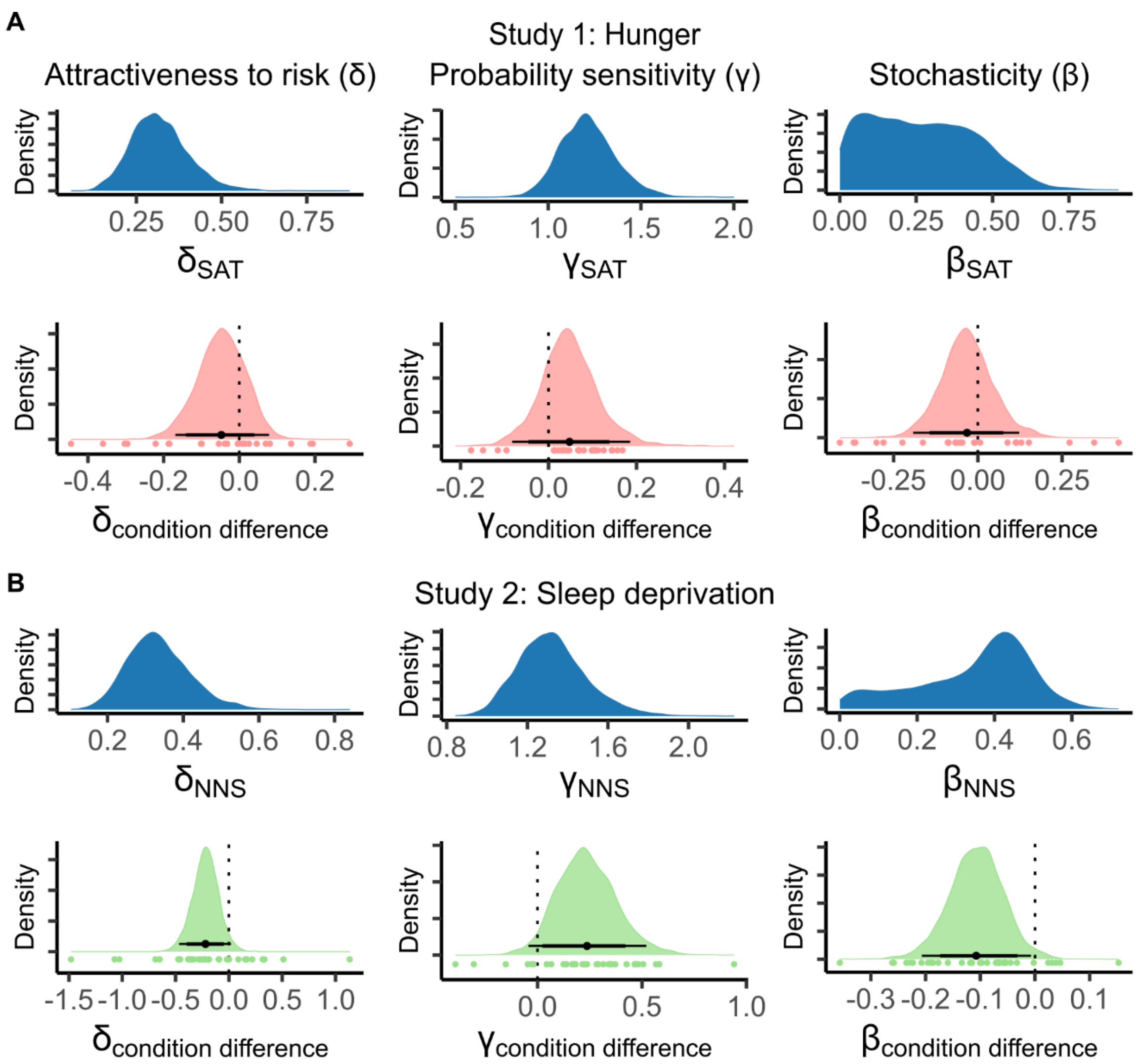

Supplementary Figure 9. Posterior Distributions of Group-Level Parameters of the Prospect Theory Model with Ghrelin Coefficients. The posterior distributions of the group-level parameters for study 1 (hunger; A) and study 2 (sleep deprivation; B): attractiveness to risk δ, sensitivity to probabilities γ, and policy parameter β. Parameter values of the sated (A) and rested (B) condition are shown in blue, the difference between conditions is shown in red for study 1 and in green for study 2. The horizontal lines represent the highest distribution interval (HDI) of 95% (thin line) and 85% (thick line), with the mean of the distribution as dot. The coloured dots underneath the distribution represent the mean values of individual subjects. Most parameters, in both studies, did not differ significantly between conditions as shown by the HDI containing zero in each difference distribution. The exception is the stochasticity in study 2. Values are reported in Supplementary Table 7 and Supplementary **Table 8**.

Neuroimaging Results

Subjective Value

Supplementary Table 9. Main Effect of Subjective Value Across Conditions. Regions with peak activity after small volumes corrections with the Rangel mask.

Region	MNI coordinates			Peak T-value	$p_{(FWE)}$
	X	Y	Z		
Study 1					
Right posterior cingulate cortex	2	−34	38	7.48	0.000
Right ventral striatum	12	11	−14	6.37	0.001
Right anterior cingulate cortex	8	42	10	6.3	0.001
Left anterior cingulate cortex	−3	42	5	5.99	0.002
Left nucleus caudatus	−12	12	2	4.86	0.027
Study 2					
Left nucleus caudatus	−9	11	4	5.43	0.003
Left anterior cingulate cortex	−3	40	8	4.91	0.01
Right posterior cingulate cortex	2	−32	34	4.53	0.027

Choice

Supplementary Table 10. Main Effect of Risky Choice versus Safe Choice. Regions with peak activity of choice after small volumes corrections with the not-thresholded choice mask from Cui and colleagues (2022).

Region	MNI coordinates			Peak T-value	$p_{(FWE)}$
	X	Y	Z		
Study 1					
Right dorsolateral prefrontal cortex	50	42	18	7.41	0.000
Right inferior parietal gyrus	50	−38	52	7.17	0.000
Right inferior temporal gyrus	60	−44	−14	7.09	0.000
Left anterior insula	−32	22	2	6.92	0.000
Right posterior cingulate cortex	0	−20	28	5.83	0.01
Study 2					
Left nucleus caudatus	−10	11	0	5.52	0.016
Left dorsolateral prefrontal cortex	−40	53	−2	5.31	0.035

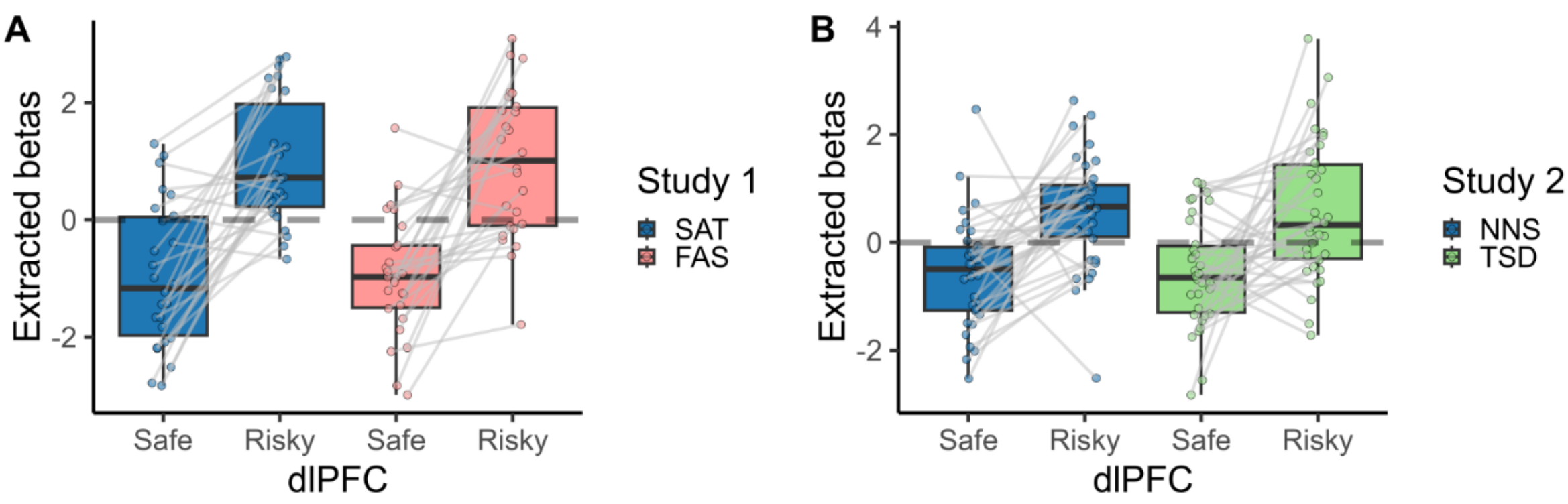

Supplementary Figure 10. Extracted β-Coefficients of Choice. The extracted β-coefficients of each participant for the peak activity in the right dlPFC (study 1, A; $X = 50, Y = 42, Z = 18$) and the left dlPFC (study 2, B; $X = -40, Y = 53, Z = -2$). While there was an effect of choice in these regions, there was no interaction with condition.

Ghrelin Correlation

Here we report supporting evidence for the lack of effect of ghrelin on the neural activity of subjective value and choice. We extracted the β-coefficients from the different GLMs (subjective value and choice) for the brain regions with significant activity and where we initially predicted to be activity related to either subjective value or choice. For the subjective value model, this was the PCC and ventral striatum in study 1, and the PCC in study 2. For the choice model, this was the dlPFC in both studies (see Supplementary Table 9 andSupplementary Table 10 for the MNI coordinates of the relevant regions for subjective value and choice respectively). The difference in β-coefficients between the experimental condition (fasted or sleep deprived) and the baseline condition (sated or normal sleep) is plotted against the difference in ghrelin levels between conditions. None of the correlations were statistically significant (see Supplementary Table 11 and Supplementary Figure 11).

Supplementary Table 11. Correlation Between Ghrelin Difference and β Difference. Pearson correlation coefficient (p-values) of the difference in ghrelin levels between conditions, and for subjective value the difference in β-coefficients between the experimental condition (fasted or sleep deprived) and the baseline condition (sated or normal sleep), for choice it is the difference in β-coefficients between the risky and safe choice.

	Study 1	Study 2
Subjective value		
Posterior cingulate cortex	$-0.35\ (.08)$	$0.15\ (.4)$
Ventral striatum	$-0.24\ (.25)$	$-$
Choice		
Dorsolateral prefrontal cortex	$0.37\ (.07)$	$0.18\ (.32)$

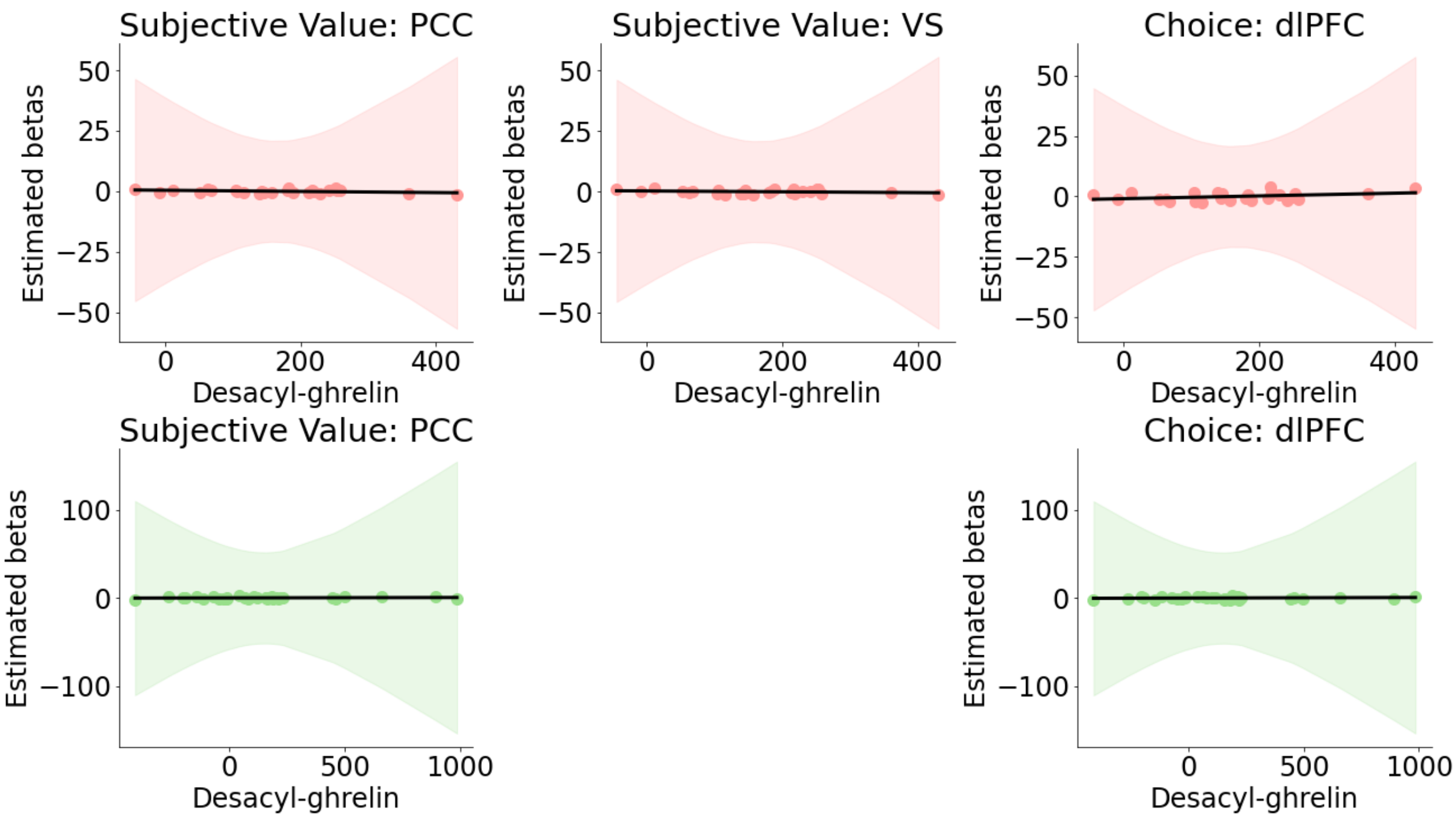

Supplementary Figure 11. Estimated β-Coefficients of fMRI Models. The difference in extracted β-coefficients between conditions of the subjective value and choice GLMs for the neuroimaging data and their relationship to the difference in ghrelin levels between conditions. The black line is the best fitting regression line with coloured confidence bounds. The correlation was never statistically significant. Study 1 (fasting) in red, study 2 (sleep deprivation) in green; posterior cingulate cortex (PCC), ventral striatum (VS), dorsolateral prefrontal cortex (dlPFC).

Insulin

Insulin is a peptide that fluctuates with levels of food intake and thus covaries with hunger. Contrary to ghrelin, there was strong evidence for a decrease in insulin levels in the fasting condition ($Mdn_{FAS} = 3.45$), compared to the sated condition ($Mdn_{SAT} = 13.15, BF > 1000 \pm 0\%$) in study 1 (Supplementary Figure 12.A). In study 2, there was anecdotal evidence against a difference between insulin levels after one night of normal sleep ($Mdn_{NNS} = 6$) and insulin levels after one night of total sleep deprivation ($Mdn_{TDS} = 5.4, BF = 0.53 \pm 0.03\%$; Supplementary Figure 12.B).

We reanalysed the difference in ghrelin levels between conditions, now by controlling for the insulin levels. For this, we compared a Bayesian linear model with insulin as a predictor for ghrelin to a model with insulin and the condition as predictive values. When controlled for insulin, there was anecdotal evidence against difference in ghrelin between both conditions in study 1 ($BF = 0.85 \pm 1.6\%$) and study 2 ($BF = 0.4 \pm 1.19\%$). This suggests that the changes in ghrelin levels were better explained by the changes in insulin levels than by the experimental manipulation.

To control for the effect of insulin in the computational model, we included insulin parameters for each model parameter. Using δ as an example and building on Equation 9 and Equation 10, the model was adjusted with

$$\delta_e = \delta_g + \delta_i = \Delta_g * \lambda_{g\delta} + \Delta_i * \lambda_{i\delta}$$

where δ_e is the sum of the ghrelin controlled parameter δ_g and the insulin controlled parameter δ_i which contains the difference in insulin between conditions Δ_i, and the insulin coefficient $\lambda_{i\delta}$ for the model's parameter, in this example δ. The same is done for γ_e and β_e. This resulted in a PT model where the subjective probability weight (Equation 2) is

$$w(P_t) = \frac{\left(\delta + c_t * (\delta_s + \delta_e)\right) * P_t^{\gamma + c_t * (\gamma_s + \gamma_e)}}{\left(\delta + c_t * (\delta_s + \delta_e)\right) * P_t^{\gamma + c_t * (\gamma_s + \gamma_e)} + (1 - P_t)^{\gamma + c_t * (\gamma_s + \gamma_e)}}$$

The model was fitted on the subset of fMRI participants. To verify that the model was able to describe the observed behaviour, we compared the simulated to the observed risky choice selection. There was a large overlap between the predicted and observed behaviour (Supplementary Figure 13).

With the inclusion of ghrelin and insulin, the model parameters did not differ between conditions according to the 95% HDI, except for β in study 2 (Supplementary Figure 14). Attractiveness to risk δ in study 2 showed a tendency towards significance but the zero was still captured by the 95% HDI. The inclusion of the covariates changed the direction of the difference between conditions, when compared to the model without covariates. For example, in study 1, the choice stochasticity decreased when controlling for insulin, while it increased when only ghrelin was included. Comparing the effects of both covariates, it is noteworthy that ghrelin and leptin affect the different parameters in opposite directions, except for γ and β in study 2. Both increase the difference of γ between conditions and decrease the difference of β between conditions.

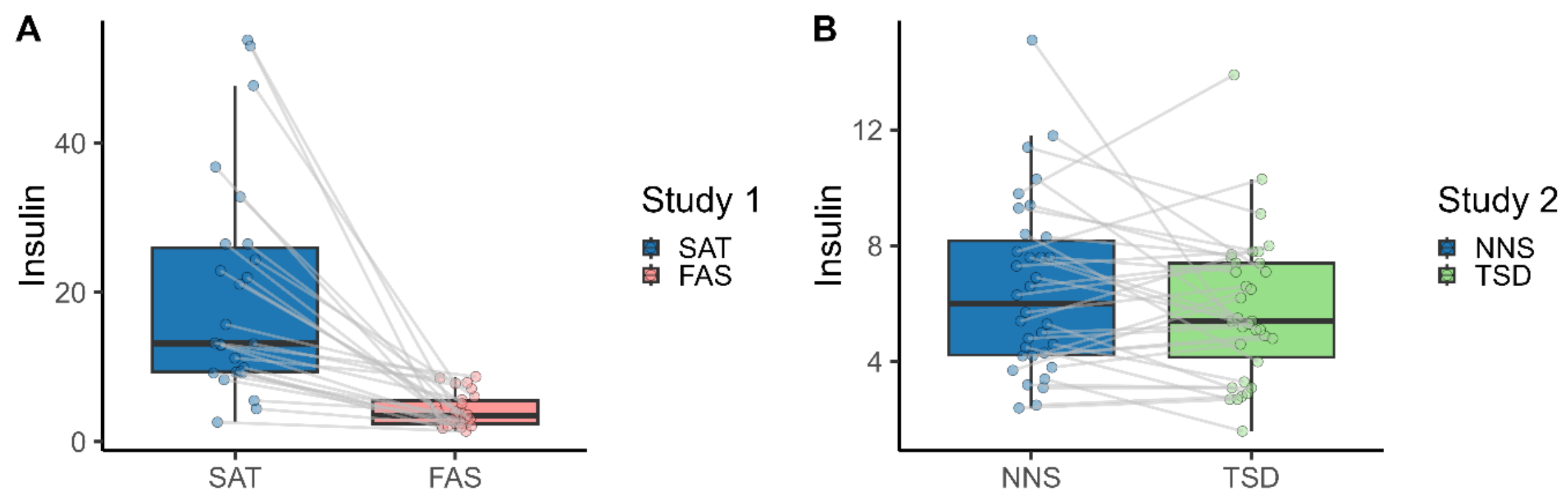

Supplementary Figure 12. Insulin Levels. A) Insulin decreased in the fasted condition (red; $Mdn_{FAS} = 3.45$) compared to the sated condition (blue; $Mdn_{SAT} = 13.15, BF > 1000 \pm 0\%$) in study 1. B) There were no changes in insulin levels between one night of normal sleep (blue; $Mdn_{NNS} = 6$) or one night of total sleep deprivation (green; $Mdn_{TSD} = 5.4, BF = 0.53 \pm 0.03\%$) in study 2.

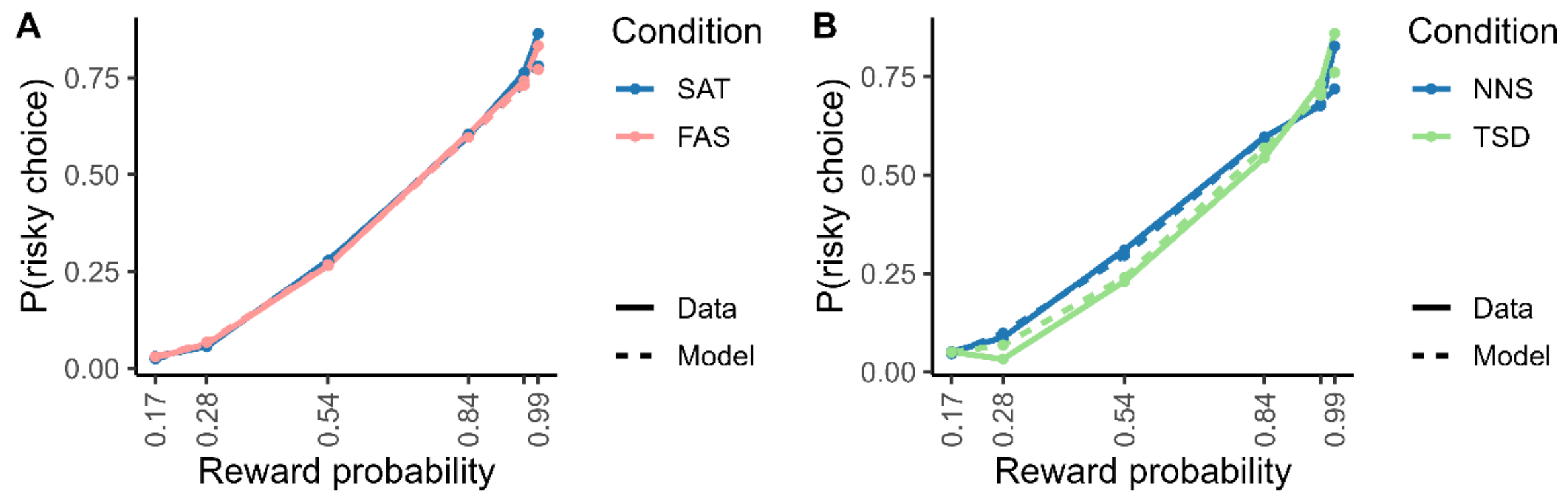

Supplementary Figure 13. Choice Predictions and Behavioural Data of the Prospect Theory Model with Ghrelin and Insulin Covariates. The probability of selecting the risky option per reward probability $(0.17, 0.28, 0.54, 0.84, 0.96, 0.99)$ for participants (full line) and simulated by the prospect theory model (dashed line). The model predictions show a large overlap with the behavioural data.

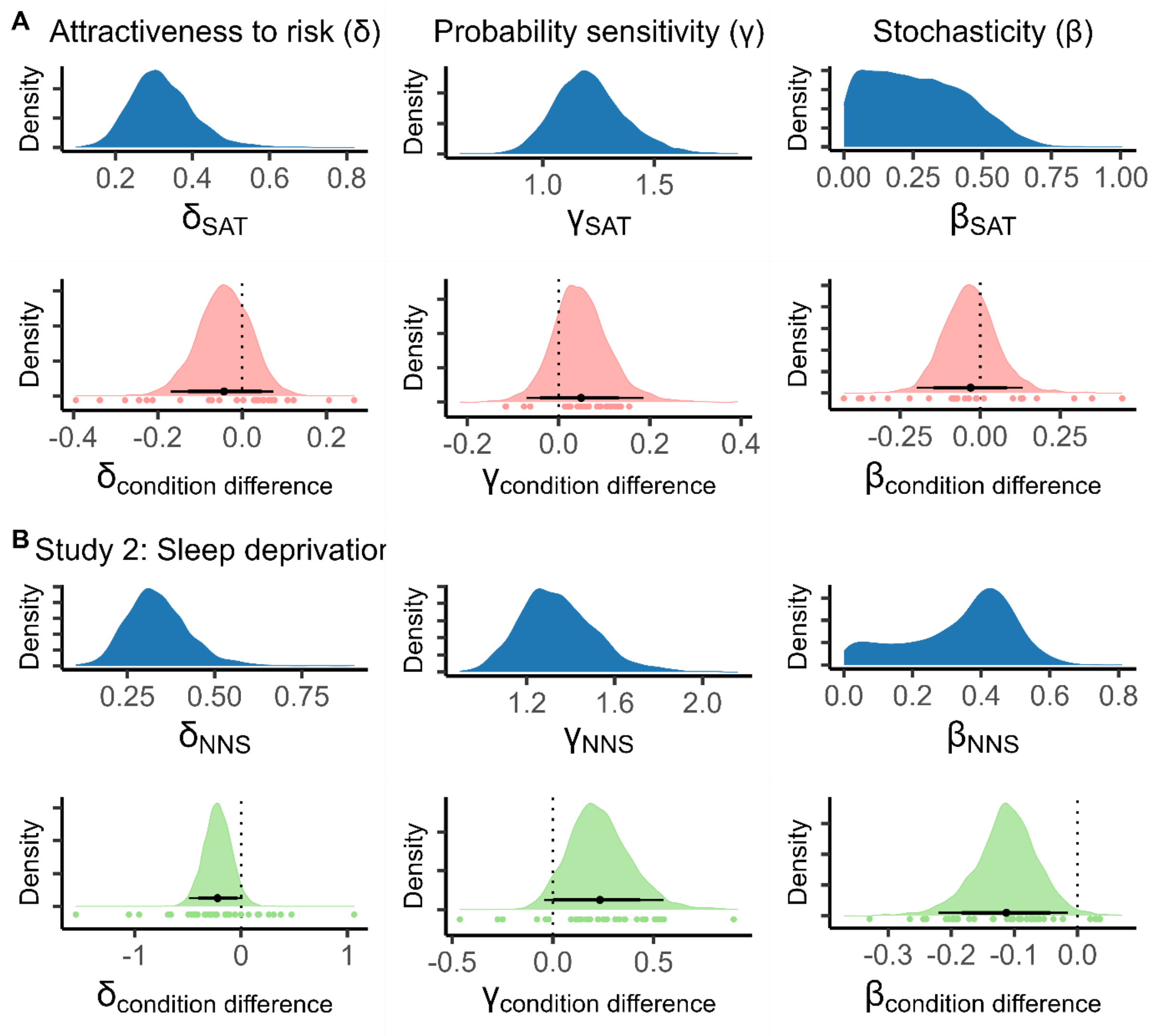

Supplementary Figure 14. Posterior Distribution of Group-Level Parameters of the Prospect Theory Model with Ghrelin and Insulin Covariates. Attractiveness to risk δ, sensitivity to probabilities γ, and policy parameter β.

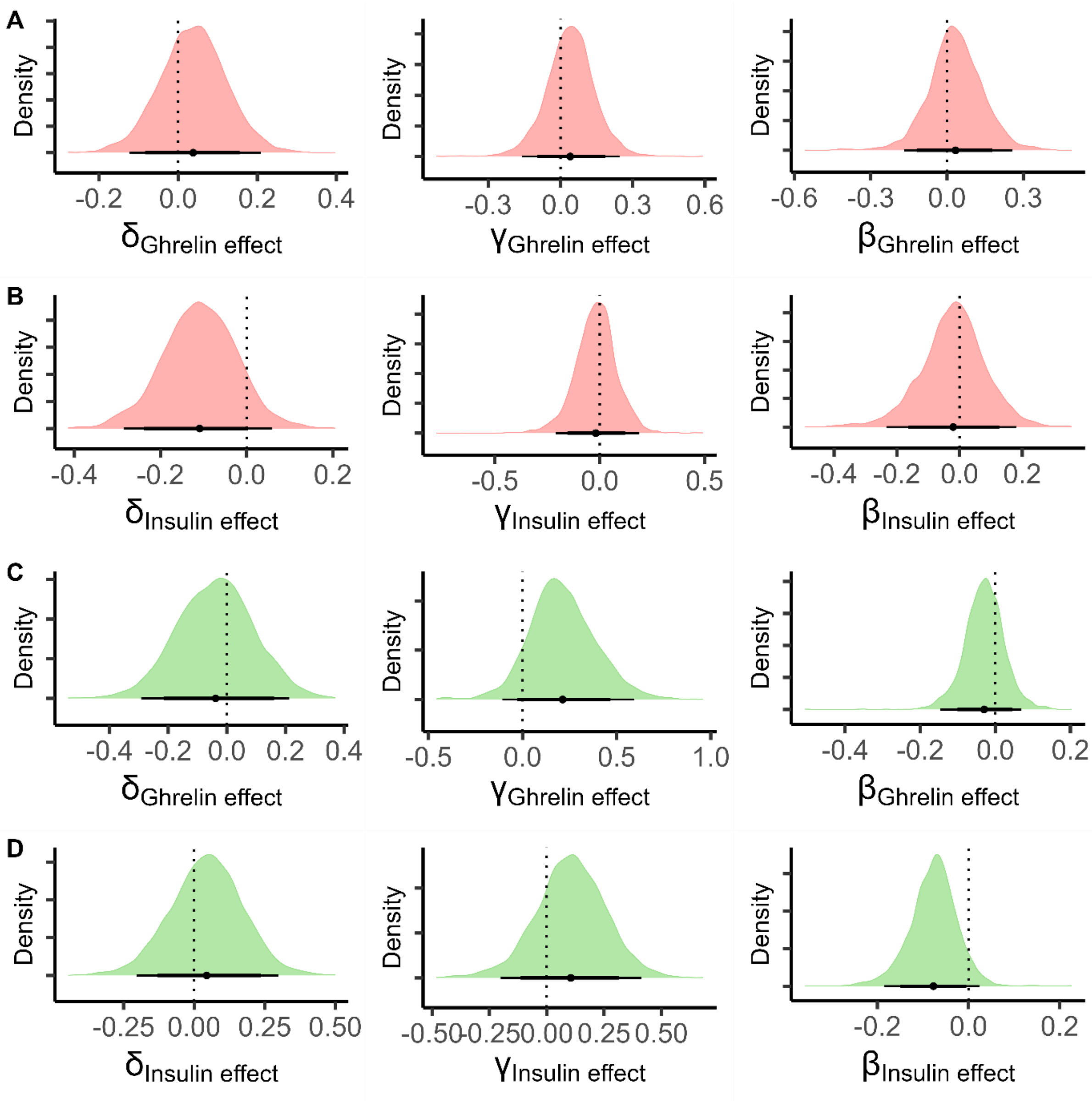

Supplementary Figure 15. Posterior Distribution of Ghrelin and Insulin Coefficients for the Parameters of the Prospect Theory Model. The posterior distributions of the ghrelin and leptin coefficients for the group-level parameters for study 1 (hunger; A and B) and study 2 (sleep deprivation; C and D): attractiveness to risk δ, sensitivity to probabilities γ, and policy parameter β. Zero falls within the highest density intervals (HDI; thick bars: 0.85, thin bars: 0.95) for all parameter in both studies.

Supplementary Table 12. Parameter Values of Prospect Theory with Ghrelin and Insulin Covariates. The mean ($\pm$SD) of the blue distributions shown in Supplementary Figure 14: the estimated parameters for the PT model for the baseline condition (i.e., sated or night of normal sleep) and the experimental condition (i.e., fasted or total sleep deprivation) at the group level. Attractiveness to risk δ, sensitivity to probabilities γ, and policy parameter β.

		Study 1	Study 2
δ	Baseline condition	$0.32\ (\pm 0.08)$	$0.34\ (\pm 0.09)$
	Experimental condition	$0.3\ (\pm 0.08)$	$0.23\ (\pm 0.08)$
γ	Baseline condition	$1.21\ (\pm 0.16)$	$1.34\ (\pm 0.17)$
	Experimental condition	$1.27\ (\pm 0.16)$	$1.612(\pm 0.23)$
β	Baseline condition	$0.27\ (\pm 0.17)$	$0.34\ (\pm 0.15)$
	Experimental condition	$0.24\ (\pm 0.19)$	$0.23\ (\pm 0.16)$

Supplementary Table 13. Condition Differences for the Prospect Theory With Ghrelin and Insulin Covariates. The difference in parameter values between the baseline and experimental condition for each study (the coloured distributions in Supplementary Figure 14).

	Mean (±SD)	95% HDI	dBF_{10}
Study 1			
Attractiveness to risk (δ)	-0.04 (± 0.06)	$-0.17, 0.07$	0.34
Sensitivity to probabilities (γ)	0.05 (± 0.06)	$-0.07, 0.19$	3.81
Policy parameter (β)	-0.03 (± 0.08)	$-0.2, 0.13$	0.52
Study 2			
Attractiveness to risk (δ)	-0.22 (± 0.13)	$-0.49, 0.01$	0.05
Sensitivity to probabilities (γ)	0.23 (± 0.16)	$-0.04, 0.55$	17.43
Policy parameter (β)	-0.11 (± 0.05)	$-0.22, -0.02$	0.01

Supplementary Table 14. Ghrelin Coefficients of the Prospect Theory Parameters. The effect of ghrelin on the estimated parameter values of the PT model presented in Supplementary Figure 15 (A and C).

	Mean ($\pm$SD)	95% HDI	dBF_{10}
Study 1			
Attractiveness to risk (δ)	$0.04\ (\pm 0.08)$	$-0.12, 0.21$	2.1
Sensitivity to probabilities (γ)	$0.04\ (\pm 0.1)$	$-0.16, 0.25$	2.04
Policy parameter (β)	$0.03\ (\pm 0.11)$	$-0.17, 0.26$	1.73
Study 2			
Attractiveness to risk (δ)	$-0.04\ (\pm 0.13)$	$-0.29, 0.21$	0.64
Sensitivity to probabilities (γ)	$0.21\ (\pm 0.18)$	$-0.11, 0.59$	9.04
Policy parameter (β)	$-0.03\ (\pm 0.05)$	$-0.15, -0.07$	0.39

Supplementary Table 15. Insulin Coefficients of the Prospect Theory Parameters. The effect of insulin on the estimated parameter values of the PT model presented in Supplementary Figure 15 (B and D).

	Mean (±SD)	95% HDI	dBF_{10}
Study 1			
Attractiveness to risk (δ)	-0.11 (± 0.09)	$-0.29, 0.06$	0.11
Sensitivity to probabilities (γ)	-0.02 (± 0.1)	$-0.21, 0.19$	0.76
Policy parameter (β)	-0.02 (± 0.1)	$-0.23, 0.18$	0.74
Study 2			
Attractiveness to risk (δ)	0.04 (± 0.13)	$-0.2, 0.3$	1.8
Sensitivity to probabilities (γ)	0.1 (± 0.15)	$-0.2, 0.41$	3.26
Policy parameter (β)	-0.08 (± 0.05)	$-0.19, -0.02$	0.06

Cortisol

Cortisol is a hormone that fluctuates with levels of food intake and thus covaries with hunger. Contrary to ghrelin, there was moderate evidence against a difference in cortisol levels between the sated ($Mdn_{SAT} = 80$) and fasted condition ($Mdn_{FAS} = 76, BF = 0.31 \pm 0.03\%$) in study 1 (Supplementary Figure 16.A). In study 2, there was anecdotal evidence against a difference between cortisol levels after one night of normal sleep ($Mdn_{NNS} = 107$) and cortisol levels after one night of total sleep deprivation ($Mdn_{TDS} = 129.5, BF = 0.64 \pm 0.03\%$; Supplementary Figure 16.B).

We reanalysed the difference in ghrelin levels between conditions, now by controlling for the cortisol levels. For this, we compared a Bayesian linear model with cortisol as a predictor for ghrelin to a model with cortisol and the condition as predictive values. When controlled for cortisol, there was very strong evidence in favour of a difference in ghrelin levels between conditions in study 1 ($BF = 67.9 \pm 0.77\%$). However, there was anecdotal evidence against a difference in ghrelin between conditions in study 2 ($BF = 0.65 \pm 1.16\%$).

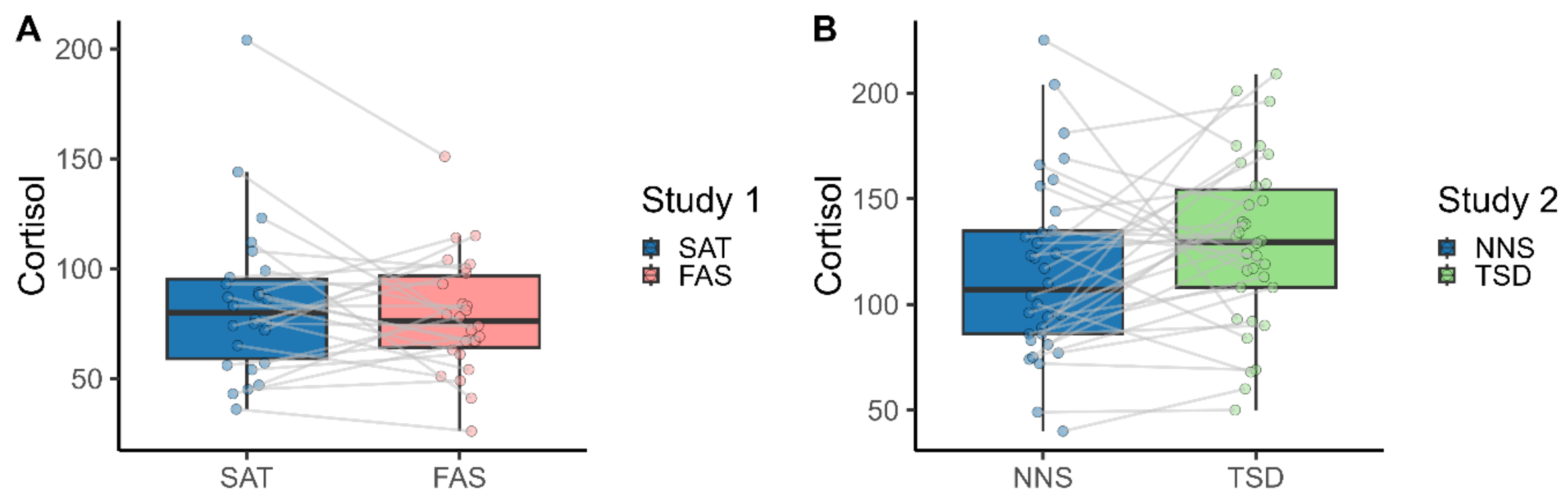

Supplementary Figure 16. Cortisol Levels. A) Cortisol did not differ between the sated ($Mdn_{SAT} = 80$) and fasted condition (red; $Mdn_{FAS} = 76, BF = 0.31 \pm 0.03\%$) in study 1. B) There were no changes in cortisol levels between one night of normal sleep (blue; $Mdn_{NNS} = 107$) or one night of total sleep deprivation (green; $Mdn_{TSD} = 129.5, BF = 0.64 \pm 0.03\%$) in study 2.

Leptin

Leptin is a hormone that fluctuates with levels of food intake and thus covaries with hunger. Contrary to ghrelin, there was strong evidence against differences in leptin between the sated ($Mdn_{SAT} = 1.66$) and fasted ($Mdn_{FAS} = 1.5, BF = 0.21 \pm 0.03\%$) condition in study 1 (Supplementary Figure 17.A). Similarly, the data in study 2 (Supplementary Figure 17.B) provided strong evidence against differences in leptin levels after one night of normal sleep ($Mdn_{NNS} = 1.48$) and leptin levels after one night of total sleep deprivation ($Mdn_{TSD} = 1.51, BF = 0.19 \pm 0.04\%$).

We reanalysed the difference in ghrelin levels between conditions, now by controlling for the leptin levels. For this, we compared a Bayesian linear model with leptin as a predictor for ghrelin to a model with leptin and the condition as predictive values. When controlled for leptin, there was very strong evidence in favour of a difference in ghrelin levels between conditions in study 1 ($BF = 59.41 \pm 1.51\%$). However, there was anecdotal evidence against a difference in ghrelin between conditions in study 2 ($BF = 0.5 \pm 0.96\%$).

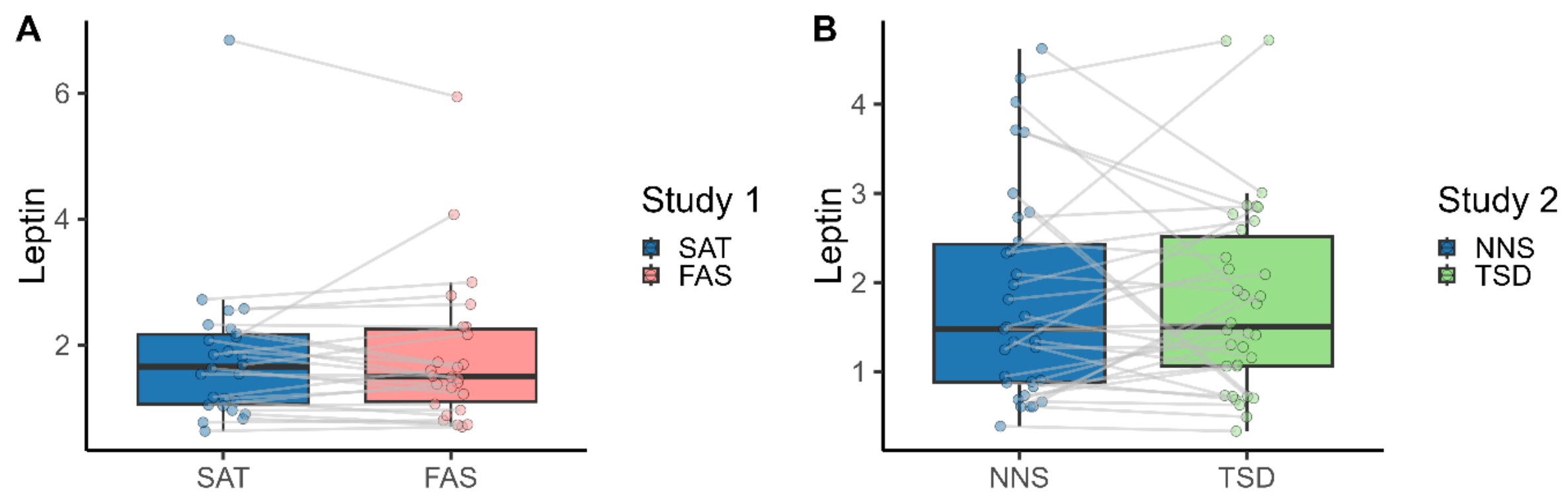

Supplementary Figure 17. Leptin Levels. The data provided strong evidence against an increase in leptin levels between sated (A, blue; $Mdn_{SAT} = 1.66$) and the fasted condition (A, red; $Mdn_{FAS} = 1.5, BF = 0.21 \pm 0.03\%$) in study 1. Similarly, in study 2, the data provided strong evidence against an increase in leptin levels after one night of total sleep deprivation (B; $Mdn_{NNS} = 1.48, Mdn_{TSD} = 1.51, BF = 0.19 \pm 0.04\%$).

Glucose

Glucose is a hormone that fluctuates with levels of food intake and thus covaries with hunger. Contrary to ghrelin, there was moderate evidence against a difference in glucose levels between the sated $(Mdn_{SAT} = 84)$ and fasted condition $(Mdn_{FAS} = 83.5, BF = 0.4 \pm 0.03\%)$ in study 1 (Supplementary Figure 18.A). In study 2, there was moderate evidence against a difference between glucose levels after one night of normal sleep $(Mdn_{NNS} = 79.5)$ and glucose levels after one night of total sleep deprivation ($Mdn_{TDS} = 78, BF = 0.19 \pm 0.04\%$; Supplementary Figure 18.B).

We reanalysed the difference in ghrelin levels between conditions, now by controlling for the glucose levels. For this, we compared a Bayesian linear model with glucose as a predictor for ghrelin to a model with glucose and the condition as predictive values. When controlled for glucose, there was strong evidence in favour of a difference in ghrelin levels between conditions in study 1 $(BF = 36.81 \pm 0.81\%)$. However, there was anecdotal evidence against a difference in ghrelin between conditions in study 2 $(BF = 0.49 \pm 1.84\%)$.

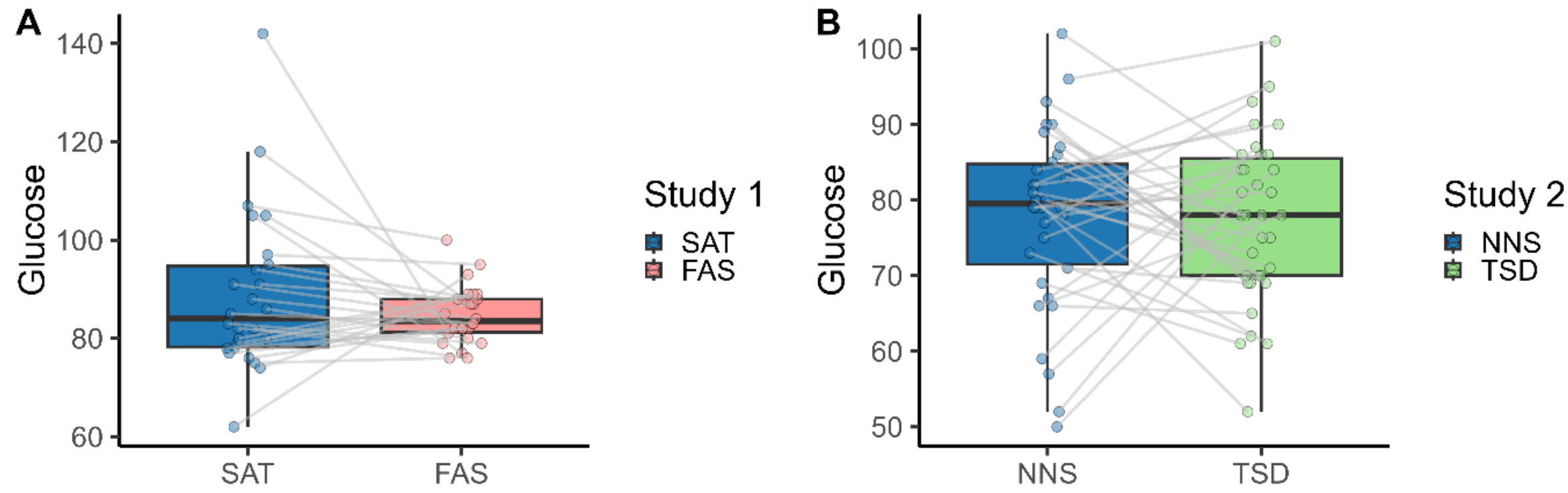

Supplementary Figure 18. Glucose Levels. A) Glucose did not differ between the sated ($Mdn_{SAT} = 84$) and fasted condition (red; $Mdn_{FAS} = 83.5, BF = 0.4 \pm 0.03\%$) in study 1. B) There were no changes in glucose levels between one night of normal sleep (blue; $Mdn_{NNS} = 79.5$) or one night of total sleep deprivation (green; $Mdn_{TSD} = 78, BF = 0.19 \pm 0.04\%$) in study 2.
